# Complementation between polymerase- and exonuclease-deficient mitochondrial DNA polymerase mutants in genomically engineered flies

**DOI:** 10.1038/ncomms9808

**Published:** 2015-11-10

**Authors:** Ana Bratic, Timo E. S. Kauppila, Bertil Macao, Sebastian Grönke, Triinu Siibak, James B. Stewart, Francesca Baggio, Jacqueline Dols, Linda Partridge, Maria Falkenberg, Anna Wredenberg, Nils-Göran Larsson

**Affiliations:** 1 Department of Mitochondrial Biology, Max Planck Institute for Biology of Ageing, Joseph-Stelzmann-Strasse 9b, Cologne D-50931, Germany; 2 Department of Medical Biochemistry and Cell Biology, Institute of Biomedicine, University of Gothenburg, Medicinaregatan 9A, Gothenburg SE-40530, Sweden; 3 Department of Biological Mechanisms of Ageing, Max Planck Institute for Biology of Ageing, Joseph-Stelzmann-Strasse 9b, Cologne D-50931, Germany; 4 Department of Laboratory Medicine, Karolinska Institutet, Stockholm SE-17177, Sweden

## Abstract

Replication errors are the main cause of mitochondrial DNA (mtDNA) mutations and a compelling approach to decrease mutation levels would therefore be to increase the fidelity of the catalytic subunit (POLγA) of the mtDNA polymerase. Here we genomically engineer the *tamas* locus, encoding fly POLγA, and introduce alleles expressing exonuclease- (exo^−^) and polymerase-deficient (pol^−^) POLγA versions. The exo^−^ mutant leads to accumulation of point mutations and linear deletions of mtDNA, whereas pol^−^ mutants cause mtDNA depletion. The mutant *tamas* alleles are developmentally lethal but can complement each other in trans resulting in viable flies with clonally expanded mtDNA mutations. Reconstitution of human mtDNA replication *in vitro* confirms that replication is a highly dynamic process where POLγA goes on and off the template to allow complementation during proofreading and elongation. The created fly models are valuable tools to study germ line transmission of mtDNA and the pathophysiology of POLγA mutation disease.

## Abstract (web summary)

A key source of mitochondrial DNA mutations is errors introduced during genome replication. Here the authors create *Drosophilia* strains with separated elongation and proofreading capabilities to explore the dynamism of mitochondrial DNA replication.

## Graphical abstract

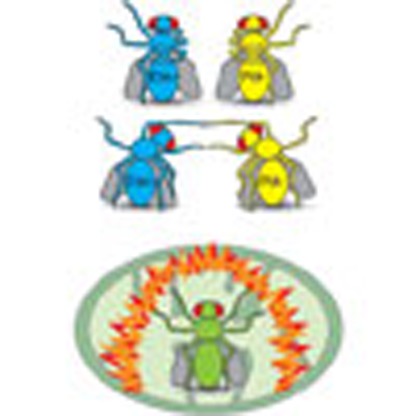

Mutations of mitochondrial DNA (mtDNA) are an important cause of human disease and are heavily implicated in the ageing process, consistent with the essential role for mtDNA in maintaining oxidative phosphorylation. MtDNA is fully coated with the mitochondrial transcription factor A (TFAM) protein and compacted into nucleoids^1^,^2^, which must be at least partly unpackaged to allow mtDNA replication^3^. The metazoan mtDNA is replicated by DNA polymerase gamma (POLγ)^4^, which belongs to the family A DNA polymerases, as does DNA polymerase I of *Escherichia coli*^5^. It consists of the catalytic (POLγA) and the accessory subunits (POLγB), which interact to form a heterodimer in insects^6^ and a heterotrimer in mammals^7^. A minimal mammalian mtDNA replisome, consisting of POLγ, TWINKLE DNA helicase and mitochondrial single-stranded DNA-binding protein (mtSSB), can synthesize DNA strands longer than 15 kb in length, thus showing that a single initiation event is sufficient to replicate one strand of mtDNA^8^. Mouse knockouts (KOs) for POLγA^9^, POLγB^10^ and TWINKLE^11^ have shown that all are essential for mtDNA maintenance and embryo survival.

The mode of mammalian mtDNA replication is debated and observations of replication intermediates by electron microscopy or two-dimensional (2D) neutral–neutral agarose gel electrophoresis have been interpreted to support either a strand-asynchronous or a strand-synchronous model^12^. The strand-asynchronous model proposes that leading (heavy (H)) strand replication initiates at a defined origin of replication (O_H_) in the control region. Once initiated the leading strand replication continues two-thirds around the circular genome until the origin of lagging (light (L)) strand replication (O_L_) is activated and lagging strand replication ensues^13^. Transcription by the mitochondrial RNA polymerase (POLRMT) at the light strand promoter provides the RNA primers necessary for initiation of replication at O_H_ and POLRMT is also responsible for providing RNA primers for initiation of replication at O_L_^14^,^15^. Consistently, KO mice lacking POLRMT develop severe mtDNA depletion causing embryonic lethality^16^. The importance of light strand promoter, O_H_ and O_L_ is further underscored by the finding that replication-competent mtDNAs, which harbour large deletions in some forms of human mitochondrial disease, always retain these sequences^17^,^18^. In addition, an *in vivo* saturation mutagenesis approach in the mouse has shown that O_L_ is indispensable for mtDNA maintenance^14^. Further support for the strand asymmetric model was recently presented in a study showing that mtSSB binds single-stranded mtDNA in a pattern consistent with initiation of replication at O_H_ and O_L_^19^.

The occurrence of mtDNA mutations has traditionally been attributed to damage^20^, but several lines of evidence suggest that replication errors may in fact be the main source of mtDNA mutations in mammals^21^,^22^,^23^ and flies^24^. Consistently, mutations in the *POLγA, POLγB* and *PEO1* (TWINKLE) genes are important causes of human disease^25^. More than 150 different pathogenic mutations have been reported in the *POLγA* gene and the majority of these causes either a severe depletion of mtDNA or the formation of large deletions. Such deletions can accumulate to high levels because mtDNA is replicated throughout the cell cycle in dividing cells and also undergoes replication in post-mitotic cells^26^. Similarly, point mutations of mtDNA can accumulate over time to high levels and affect respiratory chain function and cell viability^27^.

The mtDNA is strictly maternally inherited in mammals and transmitted without any apparent germline recombination^28^. This asexual mode of transmission is predicted to lead to accumulation of deleterious mutations over time^29^. Characterization of mtDNA sequence variation in animal models^21^,^30^,^31^,^32^, human populations^33^ and wild animals^34^ shows clear evidence of purifying selection. There is likely more than one mechanism that prevents the accumulation of mtDNA mutations between generations^29^. First, the bottleneck mechanism in the maternal mammalian germline can shift the mtDNA genotype in just a few generations and can thereby remove mutations from maternal lineages^35^,^36^,^37^,^38^,^39^. Second, there is very strong purifying selection against mutations that cause amino acid substitutions in mtDNA-encoded proteins^32^,^40^. Third, there is a selection against high levels of pathogenic mutations in transfer RNA genes post fertilization^30^. Fourth, females with high levels of mtDNA mutations in the germline have reduced fecundity^41^, which will decrease the risk of transmission of mtDNA mutations.

Given the substantial impact that mtDNA mutations have on human disease and ageing^27^, there is a strong interest in developing animal models that have increased or decreased levels of mtDNA mutations. The mtDNA mutator mice are homozygous for a knock-in mutation that leads to the expression of POLγA with a severely impaired proofreading during mtDNA replication. These mice develop a premature ageing syndrome and have a shortened life span^42^,^43^. Furthermore, mouse strains containing only a subset of all different mtDNA mutations present in mtDNA mutator mice can be derived by breeding and are valuable tools to study mtDNA transmission in the germline^21^,^30^,^41^. While mutator variants with decreased accuracy have been generated for many different DNA polymerases, only a handful of alterations are known to make DNA polymerases more accurate. High-fidelity DNA polymerases have been studied in unicellular organisms, for example, DNA polymerase I in *E. coli*^44^ and MIP1 (homolog of POLγA) in *Saccharomyces cerevisiae*^45^, but not yet in metazoans. A critical question concerning the role for mtDNA mutations in ageing is to determine whether a decreased mtDNA mutation rate would prolong life span. It is still elusive whether it is possible to engineer enzymes surpassing the accuracy of wild-type (WT) metazoan POLγA *in vivo*. To address this issue, we performed a detailed biochemical characterization of exonuclease-deficient (exo^−^, low fidelity) and polymerase-deficient (pol^−^, high fidelity) variants of human POLγA (HsPOLγA) *in vitro* and created genomically engineered fruit flies expressing the corresponding alleles of fruit fly POLγA (DmPOLγA). Homozygous expression of an exo^−^DmPOLγA variant in flies leads to accumulation of point mutations and linear deletions of mtDNA, whereas the high-fidelity variants cause mtDNA depletion, which in both situations result in larval lethality. Unexpectedly, exo^−^ and pol^−^ versions of POLγA complemented each other in trans, resulting in apparently normal compound heterozygous flies with clonally expanded mtDNA mutations. Reconstitution of human mtDNA replication *in vitro* confirmed that mtDNA replication is a highly dynamic process where HsPOLγA goes on and off the template to allow complementation during proofreading and elongation.

## Results

### HsPOLγA pol^−^ mutants have increased exonuclease activity

Two amino acid substitutions (Q849A and H881A) in *E. coli* DNA polymerase I are known to cause an antimutator effect during DNA replication^44^. As both of these amino acids are highly conserved in family A polymerases and thus present in both DmPOLγA (Q1009A and H1038A) and HsPOLγA (Q1102A and H1134A; Supplementary Fig. 1), we engineered recombinant DmPOLγA and HsPOLγA proteins and tested their effects on DNA replication *in vitro*. In addition, we chose to introduce a mutation affecting the exonuclease domain of POLγA (DmD263A, HsD274A) because such a substitution has previously been shown to exert a mtDNA mutator phenotype in budding yeast^46^ and mice^42^,^43^.

**Figure 1:**
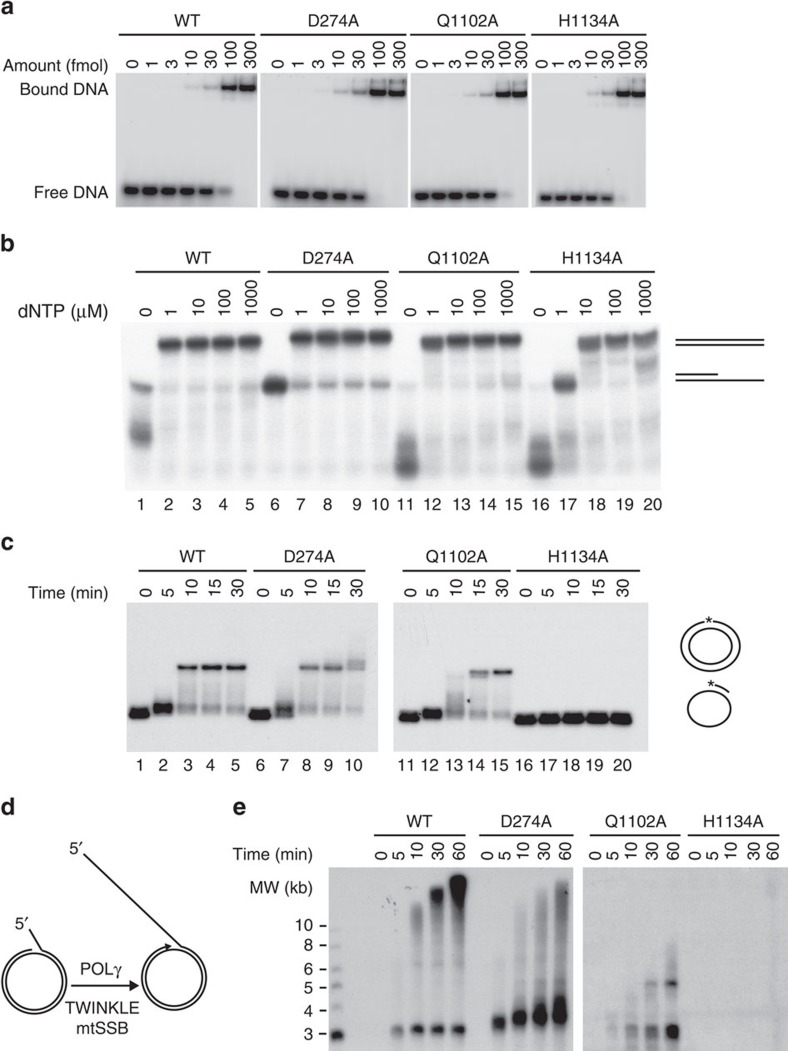
*In vitro* characterization of HsPOLγA D274A, Q1102A and H1134A mutant proteins. (**a**) Representative gels showing electrophoretic mobility assays using WT and mutant HsPOLγ holoenzymes (HsPOLγA/HsPOLγB in complex) to estimate the Kd (DNA) values. Each lane contains 10 fmol of DNA substrate and the indicated amounts of protein complex. Values of dissociation constants presented as an average from three independent binding assays are shown on Table 1. (**b**) Coupled exonuclease-polymerase assay. Using the same template as in ‘**a**' but in the presence of increasing amounts of dNTPs, H1134A required higher dNTP concentrations than the other HsPOLγA variants to polymerize the 35-mer. Reactions were run on a denaturing 15% PAGE. (**c**) Second strand synthesis. Q1102A was a slower DNA polymerase than the WT and D274A proteins, whereas H1134A was not able to produce long stretches of DNA. The template consisted of a 5′ radioactively labelled (asterisk represents the labelling) 32-mer hybridized to a single-stranded pBluescript DNA. (**d**) Schematic representation of the rolling circle *in vitro* replication assay used to analyse the function of different HsPOLγA variants in the context of the minimal mitochondrial replisome. In the presence of mtSSB and the TWINKLE helicase, POLγ is able to synthesize long stretches of DNA. (**e**) Rolling circle *in vitro* replication assay. The time curve shows the inability of the H1134A variant to produce long stretches of DNA also in the context of the replisome. Also Q1102A had also problems in generating long products. All experiments were repeated at least three times.

Human recombinant HsPOLγA proteins were purified to near homogeneity after expression in insect cells to obtain WT, D274A, Q1102A and H1134A variants (Supplementary Fig. 2a). Unfortunately, despite several attempts we were not able to purify stable and enzymatically active recombinant DmPOLγA. Therefore all of the *in vitro* experiments were performed using the recombinant human proteins. We first tested the ability of these recombinant HsPOLγA proteins to bind DNA in the presence of the processivity factor HsPOLγB by using a primer-template and standard electrophoretic mobility shift assay (EMSA) (Supplementary Fig. 2b). All HsPOLγA mutants bound DNA and interacted with HsPOLγB in a manner indistinguishable from the WT enzyme (Supplementary Fig. 2b). We also determined the Kd for DNA binding for all of the HsPOLγ mutants and found values that were very similar to the Kd for the WT enzyme (Fig. 1a and Table 1).

The WT HsPOLγA has an exonuclease activity and will engage in 3′ to 5′ degradation of DNA in the absence of deoxynucleotide (dNTP)s (Fig. 1b, lane 1), whereas it exerts polymerase activity in the presence of dNTPs (Fig. 1b, lanes 2–5). The recombinant D274A protein displayed severely reduced exonuclease activity (Fig. 1b, lane 6), while maintaining polymerase activity (Fig. 1b, lane 7–10). In contrast, the Q1102A and H1134A variants had more pronounced exonuclease activities (Fig. 1b, lanes 11 and 16) compared with the WT protein (Fig. 1b, lane 1), indicating increased fidelity^44^. While the Q1102A variant retained a polymerase activity (Fig. 1b, lanes 12–15) similar to the WT enzyme on a short template (Fig. 1b, lanes 2–5), the H1134A mutant protein required higher dNTP concentrations to synthesize DNA (Fig. 1b, lanes 17–20). We additionally tested whether any of the HsPOLγA mutant variants were capable of synthesizing long stretches of DNA using a circular ssDNA template. The D274A variant fully replicated circular ssDNA with a similar efficiency as the WT polymerase (Fig. 1c). The Q1102A variant was also able to synthesize a full-length product, *albeit* at a slower rate (Fig. 1c lanes 11–15), whereas the H1134A variant was unable to replicate DNA under these assay conditions (Fig. 1c, lanes 16–20). The low polymerase activity of the two pol^−^ variants was also verified in a processivity assay and the defect was again more severe for the H1134A mutant than for the Q1102A mutant (Supplementary Fig. 2c). In competition assays, we found that the H1134A mutant had some dominant negative effects (Supplementary Fig. 2d). We also tested whether the HsPOLγA variants could initiate DNA replication *in vitro* by performing rolling circle replication assays (Fig. 1d)^15^. Both WT and D274A enzymes generated DNA products of ∼20 kb in the presence of the mitochondrial replicative helicase TWINKLE, although D274A was a bit less efficient (Fig. 1e). In contrast, the pol^−^ mutations Q1102A and H1134A failed to synthesize long DNA products (Fig. 1e). The *in vitro* DNA replication results thus show that the D274A variant of HsPOLγA has reduced exonuclease and high polymerase activities, whereas the Q1102A and H1134A variants have increased exonuclease and reduced polymerase activities.

### DmPOLγA (tamas) KO flies show developmental lethality

**Figure 2:**
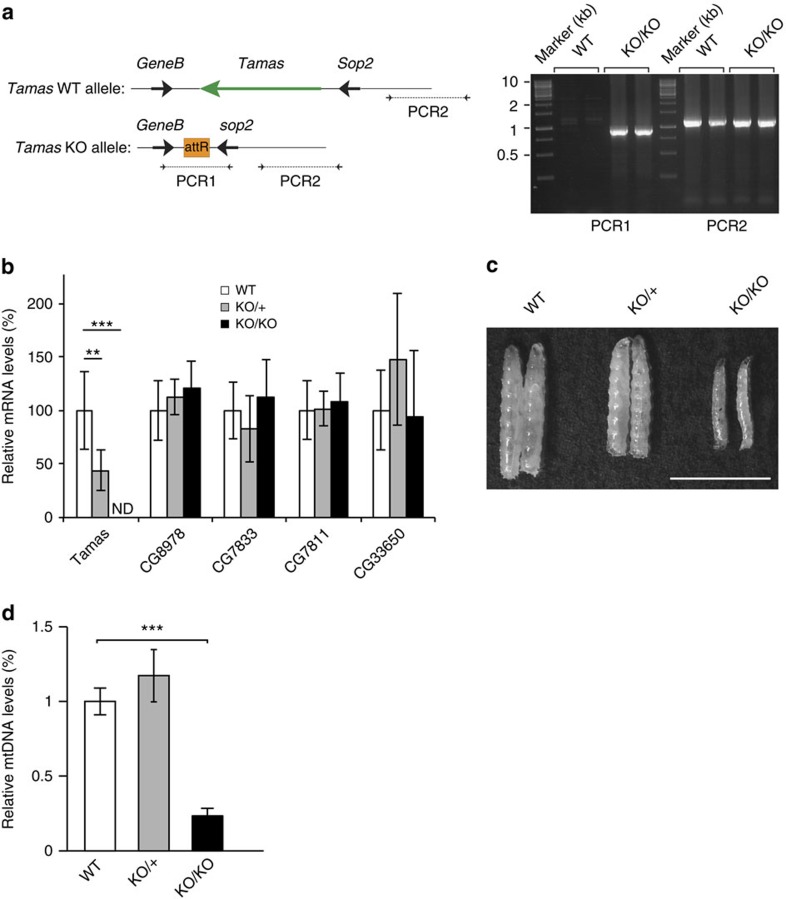
Creation of a DmPOLγA knockout founder line by using genomic engineering. (**a**) The *DmPOLγA* (*tamas*) locus and generation of *DmPOLγA* knockout (KO) mutant. *Tamas* gene encoding DmPOLγA is located on the second chromosome at cytological position 34D1. The *DmPOLγA* knockout founder fly line was generated by ends-out homologous recombination. Deletion of the *tamas* gene was confirmed by PCR1, while PCR2 was used as a control. Total DNA was extracted from *DmPOLγA* knockout larvae (KO) and wild-type (WT) flies. (**b**) RNA expression levels of *tamas* and the flanking genes in the *tamas* knockout larvae (KO). The expression of DmPOLγA (*tamas)* gene was not detected (ND) in the homozygous (KO/KO, black bar) knockout larvae. The expression of *DmPOLγA* (*tamas)* neighbouring genes was not affected by removal of *tamas* allele in *DmPOLγA* heterozygous (KO/+, grey bar) and homozygous (KO/KO, black bar) knockout larvae. Total RNA was extracted from 5-day-old larvae and gene expression was analysed by qRT–PCR. One-way ANOVA with Dunnett's *post hoc* test. ***P<0.001, **P<0.01. Error bars represent s.d. *n*=5. Data represent two independent experiments. (**c**) Comparison of body size between wild-type (WT, white bar), heterozygous (KO/+, grey bar) and homozygous *DmPOLγA* knockout (KO/KO, black bar) larvae. Homozygous knockout larvae were smaller than the wild-type and heterozygous knockout larvae. Scale bar, 5 mm. (**d**) Steady-state levels of mtDNA were determined by quantitative PCR in 5-day-old wild-type (WT, white bar), heterozygous (KO/+, grey bar) and homozygous (KO/KO, black bar) *DmPOLγA* knockout larvae. Lack of DmPOLγA led to severe mtDNA depletion. Data represent at least three independent experiments. Kruskal–Wallis test with Dunnett's *post hoc* test. ***P<0.001. Error bars represent s.d. *n*=4–6.

We next proceeded to generate a founder line to allow subsequent introduction of mutant alleles into the endogenous tamas locus by using genomic engineering. This strategy ensures that the expression of the mutant DmPOLγA variants is regulated in a physiological way, which is important because previous studies have shown that forced expression of DmPOLγA in flies results in mtDNA depletion and lethality likely caused by an imbalance in the levels of DmPOLγA and DmPOLγB^47^. To this end, we first generated DmPOLγA KO flies by replacing the tamas gene with a short attP site after 'ends-out' homologous recombination (Supplementary Fig. 3a)^48^. Precise excision of the tamas gene and insertion of the attP site was verified by PCR and sequencing (Fig. 2a and Supplementary Fig. 3b). The tamas KO larvae contained no detectable tamas mRNA whereas the mRNA expression from neighboring genes was normal (Fig. 2b). The KO of tamas was thus efficient and the presence of the attP sequence does not disturb the expression of the adjacent genes. Heterozygous DmPOLγA KO flies (KO/+) developed normally to adulthood, whereas intercrosses produced no homozygous KO (KO/KO) flies, thus showing that DmPOLγA is essential for fly survival. To show that the lethality phenotype is caused by loss of tamas, we performed genetic complementation tests between tamas KO mutants and deficiency lines that cover the locus (Supplementary Fig. 3d and Supplementary Table 1). The deficiencies that cover the tamas locus could not rescue the phenotype, whereas a deficiency adjacent to the tamas locus fully rescued the tamas KO mutant (Supplementary Table 1). The different deficiencies could not complement each other (Supplementary Table 1). The homozygous tamas KO larvae (KO/KO) died at the third instar (L3) stage (Fig. 2c) with a severe reduction in body weight (Supplementary Fig. 3c) and a 70% decrease in mtDNA levels (Fig. 2d). The residual mtDNA levels in the tamas KO/KO larvae are most likely explained by maternal contribution of both mtDNA and DmPOLγA to the embryo, which allows the homozygous KOs to develop until the L3 stage. A similar maternal contribution is well-documented in mammals, where mouse embryos with severe mtDNA replication defects survive until after implantation^9^,^11^,^49^, and in *Caenorhabditis elegans*, where loss of POLγ leads to apparently normal development albeit with severely reduced gonadal function^50^.

### Homozygous DmPOLγA exo^−^ and pol^−^ flies arrest in development

**Figure 3:**
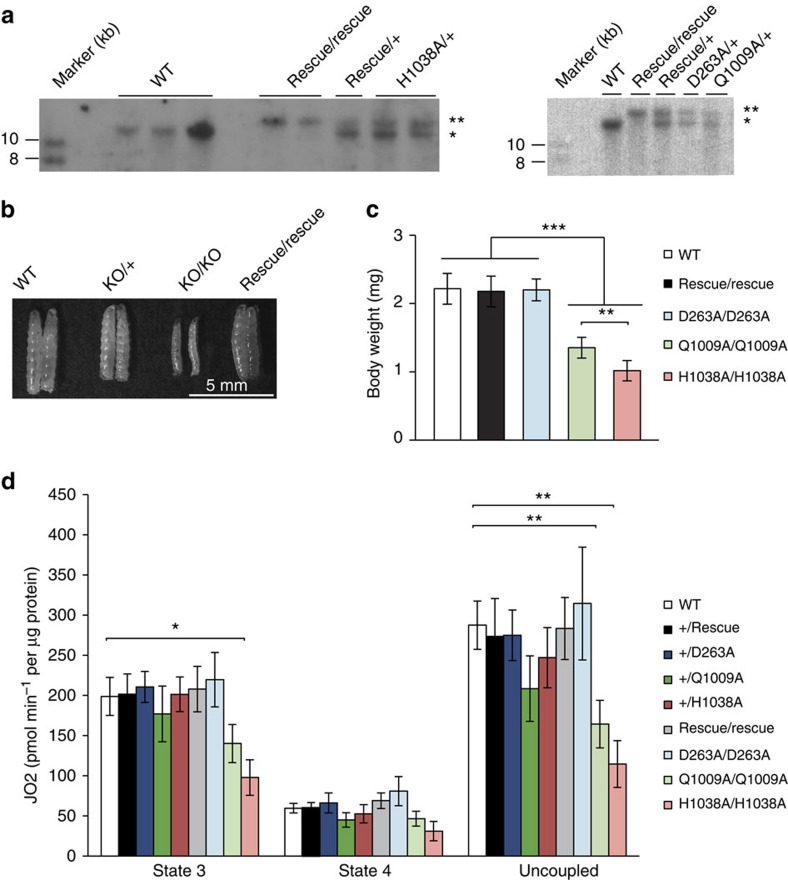
Phenotypical characterization of the genomically engineered DmPOLγA exo^−^ and pol^−^ flies. (**a**) Verification of precise re-integration of *tamas* allelic variants by Southern blot analysis. Restriction of genomic DNA with XhoI generated a ∼12.1 kb band in wild-type allele (one asterisk) and ∼15 kb band in the mutant *DmPOLγA* alleles ('**' indicate replacement alleles: Rescue, D263A, Q1009A and H1038A). A *DmPOLγA* cDNA was used as a probe. Total DNA was extracted from adult flies. (**b**) Comparison of body size between wild-type, heterozygous (KO/+) and homozygous *DmPOLγA* knockout (KO/KO) and homozygous rescue larvae. Re-introduction of the WT allele (rescue) to the genomically engineered *DmPOLγA* locus rescued the phenotypes of the knockout larvae. Scale bar, 5 mm. (**c**) Quantification of the body weight of genomically engineered *DmPOLγA* homozygous larvae. Homozygous DmPOLγA Q1009A and H1038A larvae were significantly smaller than the wild-type (WT), rescue and D263A larvae. Tukey's Multiple Comparison Test. ***P<0.001, **P<0.01. Error bars represent s.d. *n*=20. All indicated genotypes correspond to 5-day-old homozygous larvae. (**d**) Respiratory chain function. Oxygen consumption rates of 5-day-old larvae were measured under phosphorylating (state 3), non-phosphorylating (state 4) and uncoupled conditions and normalized to total protein content. Data represent two to three independent experiments. Mann–Whitney test, two-tailed. **P<0.01, *P<0.05. Error bars represent s.d. *n*=4.

POLγA is highly conserved (Supplementary Fig. 1) and the human and fly orthologues have 56.1% amino acid identity (http://mobyle.pasteur.fr). The residues D274, Q1102 and H1134 of HsPOLγA correspond to residues D263, Q1009 and H1038 of DmPOLγA, respectively (Supplementary Fig. 1). We utilized site-specific integration and the attP site^48^ to introduce mutant alleles into the tamas locus (Supplementary Fig. 3a) and generated flies expressing the D263A, Q1009A or H1038A variants of DmPOLγA (Supplementary Fig. 4a–d). For simplicity, we hereafter refer to the corresponding alleles as the D263A, Q1009A and H1038A alleles, respectively. As a control, we also introduced WT DmPOLγA to rescue the KO allele. Correct re-integration of the introduced tamas alleles was confirmed by Southern blot and PCR analyses (Fig. 3a, Supplementary Fig. 4a). Reintroduction of WT DmPOLγA (rescue allele) resulted in apparently normal homozygous flies, which demonstrate that the lethality of tamas KO flies is caused by the absence of DmPOLγA (Fig. 3b). In addition, the rescue allele had no or very mild impact on the expression of neighbouring genes in 5-day-old rescue larvae (L3 stage), thus excluding positional effects in the targeted region (Supplementary Fig. 4c). Larvae homozygous for the D263A, Q1009A or H1038A alleles died mostly at the late L3 stage (Supplementary Fig. 4d and Supplementary Table 2) although some D263A larvae entered the pupal stage and died shortly thereafter (Supplementary Table 4). Body size and weight of homozygous rescue or D263A larvae were comparable to WT larvae, while H1038A and Q1009A mutants were significantly smaller (Fig. 3c and Supplementary Fig. 4d). Furthermore, excision of the mini-white marker (Supplementary Fig. 4b) did not affect the survival or visible phenotypes of the genomically engineered flies, which shows that the observed phenotypes are a direct consequence of the introduced mutant variants of DmPOLγA.

The oxygen consumption rates in permeabilized tissues were normal in 5-day-old homozygous rescue larvae (Fig. 3d). The homozygous D263A larvae did not display any detectable changes in state 3 or state 4 respiration (Fig. 3d), whereas the homozygous Q1009A or H1038A larvae showed a profound reduction in both state 3 and uncoupled respiration (Fig. 3d).

The rescue, D263A, Q1009A and H1038A alleles were crossed over the deficiencies described above as well as over the *tam3* and *tam4* hypomorphic *POLγA* alleles^51^. We only obtained complementation with the rescue allele and it complemented all of the deficiencies covering the *tamas* locus, as well as the *tam3* and *tam4* hypomorphic alleles (Supplementary Table 3). Developmental assays were performed with the exo^−^ and pol^−^ *tamas* alleles crossed over the *tamas* KO allele to detect any possible antimorphic effects (Supplementary Table 4). The results were strongly dependent on whether the mutant allele was transmitted maternally or paternally, and we therefore performed the crosses in both directions. In all studied combinations, the homozygous *tamas* KO allele had the most severe developmental lethality phenotype (Supplementary Table 4). The homozygous D263A exo^−^ flies died in the early pupal stage, whereas hemizygous flies with paternal (KO/D263A) or maternal (D263A/KO) transmission of the D263A allele generated sick escapers that reached the adult stage. The homozygous pol^−^ flies died before pupariation, whereas the hemizygous pol^−^ flies with a paternally transmitted mutant allele (KO/H1038A and KO/Q1009A) reached the pupal stage. The larval body weight (Supplementary Fig. 5a) and the mtDNA levels (Supplementary Fig. 5b) correlated with the developmental phenotype. However, the H1038A allele showed a different pattern as these larvae had the same low body mass as the KO larvae, but less mtDNA than KO larvae (Supplementary Fig. 5a,b). The H1038A mutant thus had some dominant negative effects, which is consistent with the *in vitro* biochemistry results of the corresponding HsPOLγA mutant (H1134A; Supplementary Fig. 2d).

### DmPOLγ exo^−^ mainly introduces transition mutations of mtDNA

**Figure 4:**
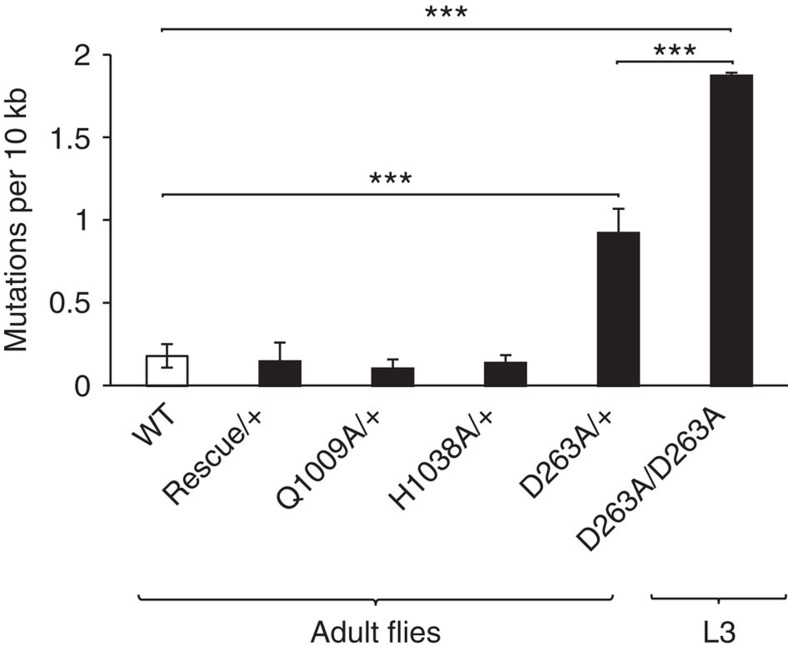
Quantitative assessment of mtDNA mutations in larvae and flies carrying different *DmPOLγA* alleles. Post-PCR cloning and sequencing was used to quantify mtDNA mutation load in young adult wild-type (WT), genomically engineered heterozygous *DmPOLγA* flies (Rescue/+, Q1009A/+, H1038A/+, D263A/+) and homozygous exo^−^ larvae (D263A/D263A). If no mutations were detected, the error rate of our method (3.5E−06) was used for further analysis. Bars represent the average number of unique mtDNA mutations. All flies inherited the mutated *DmPOLγA* allele maternally, hence the detected mtDNA mutations represent both inherited and somatic mutations. One-way ANOVA with Tukey's test. ***P<0.001. Error bars represent s.d. *n*=3–4.

We performed extensive cloning and sequencing to quantify the mtDNA mutation loads in the three mutated fly lines, as well as in the rescue line. The mtDNA mutation levels were low in WT flies (∼10^−5^ mutations per bp), consistent with a previous report^24^. In contrast, the heterozygous D263A mutant flies had significantly increased mtDNA mutation levels with on an average 7 × 10^−5^ mutations per bp (Fig. 4). In 5-day-old larvae homozygous for the D263A mutant allele (mtDNA mutator larvae), a further increase of the mtDNA mutation load (∼2 × 10^−4^ mutations per bp) was observed (Fig. 4). The heterozygous D263A mutant flies carried more transition mutations (purine>purine or pyrimidine>pyrimidine) than transversions (purine>pyrimidine or pyrimidine>purine), and all possible transitions occurred at an equal frequency (Table 2). In contrast, the T>A transversions (relative to the reference sequence) were much more frequent than the sum of all other transversion mutations (Table 2). Heterozygous flies carrying the Q1009A or H1038A high-fidelity alleles had mtDNA point mutation loads similar to the controls (Fig. 4).

### Linear mtDNA deletions in homozygous DmPOLγA exo^−^ larvae

**Figure 5:**
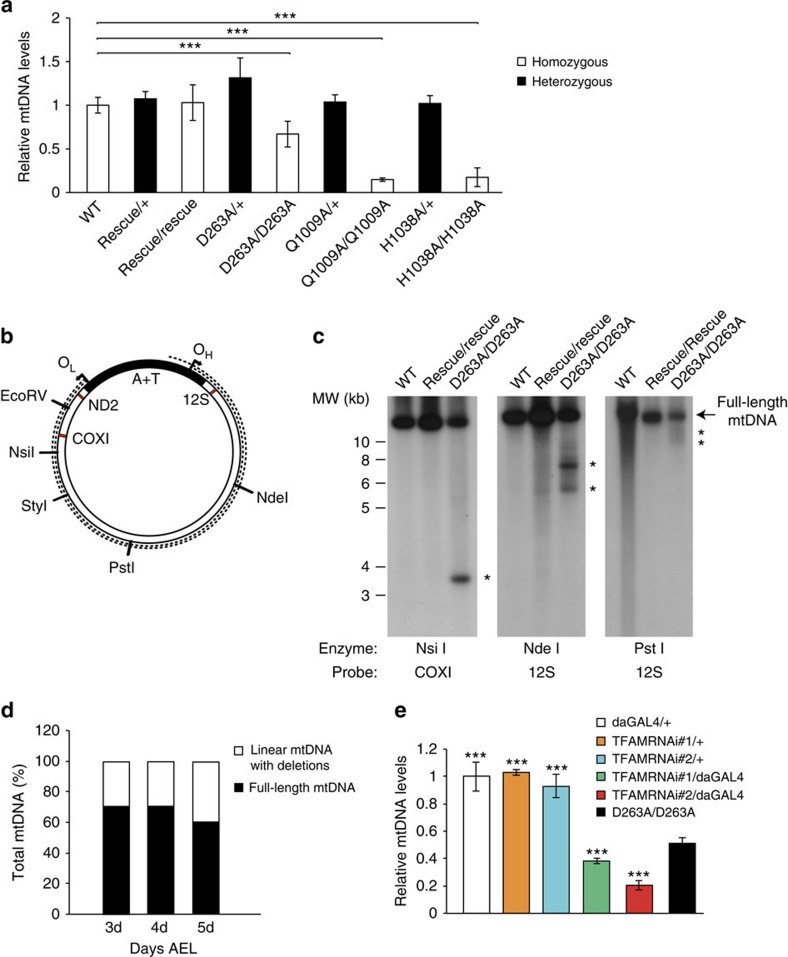
Quantification of mtDNA copy number and analysis of mtDNA integrity. (**a**) Quantification of mtDNA levels in genomically engineered DmPOLγA flies. Steady-state levels of mtDNA were determined by quantitative PCR in 5-day-old homozygous (white bar) and heterozygous (black bar) larvae. Data represent two to four independent experiments. Kruskal–Wallis test with Dunnett's *post hoc* test. ***P<0.001. Error bars represent s.d. *n*=4–6. (**b**) Schematic representation of *Drosophila* mtDNA. The dotted lines indicate linear mtDNA molecules with deletions. Restriction endonucleases used for mapping the deletions in the mtDNA are indicated. Red bars represent oligonucleotide probes used to map the mtDNA deletions. O_H_ (origin of replication for heavy strand of mtDNA) and O_L_ (origin of replication for light strand of mtDNA), A+T (control region of fly mtDNA). (**c**) Mapping mtDNA deletions. MtDNA was cut with NsiI (left panel), NdeI (middle panel) or PstI (right panel). MtDNA deletions were mapped with COXI and 12S rRNA probes. (**d**) The relative amount of mtDNA deletions increases throughout the larval development in homozygous DmPOLγA D263A mutants. Total DNA was extracted from 3-day-, 4-day- and 5-day-old homozygous D263A exo^−^ larvae and restricted with StyI. MtDNA deletions were detected using a COXI probe. Data represent two independent experiments. (**e**) Ubiquitous TFAM knockdown leads to severe mtDNA depletion. TFAM knockdown lead to a more pronounced decrease in mtDNA copy number in comparison with homozygous exo^−^ larvae. All genotypes were compared against the homozygous exo^−^ larvae (D263A/D263A). Data represent two independent experiments. One-way ANOVA with Dunnett's *post hoc* test. ***P<0.001. Error bars represent s.d. *n*=5.

We performed quantitative PCR (qPCR) and Southern blot analyses of larvae heterozygous for the D263A, Q1009A and H1038A alleles and found no significant changes in mtDNA steady-state levels in comparison with WT larvae (Fig. 5a). Larvae homozygous for the D263A allele displayed a mild reduction in mtDNA levels (Fig. 5a), whereas larvae homozygous for the Q1009A and H1038A alleles showed severe mtDNA depletion (Fig. 5a), which is consistent with the *in vitro* biochemistry results (Fig. 1, Supplementary Fig. 2c,d).

Interestingly, Southern blot analysis revealed the presence of high levels of two species of linear mtDNA molecules with deletions in larvae homozygous for the D263A allele (Fig. 5b,c). These aberrant molecules were not observed in the heterozygous D263A larvae (Supplementary Fig. 6a). Detailed mapping of the linear mtDNAs with deletions revealed two species that differed by ∼1 kb in size (Fig. 5c). The ends of the linear mtDNAs with deletions reach the A+T-rich control region, which have been suggested to contain both the O_H_ and O_L_ for fly mtDNA (Fig. 5b)^52^. The linear mtDNA with deletions already appears in the second instar stage (3 days after egg laying (AEL)), and their levels reach ∼40% of total mtDNA at 5 days AEL (Fig. 5d, Supplementary Fig. 6b), meaning that the homozygous D263A mutant larvae have a deficiency of full-length mtDNA (Fig. 5d). Southern blot analysis of the polymerase domain mutants, Q1009A and H1038A revealed no deletions (Supplementary Fig. 6c), demonstrating that the exonuclease-deficient DmPOLγA is directly involved in generating the linear mtDNA deletions.

To determine whether the homozygous D263A larvae are dying because of depletion of full-length mtDNA molecules, we determined the amount of mtDNA needed to enter and go through the pupariation stage. To this end, we characterized, molecularly and phenotypically, two independent TFAM knockdown lines, as TFAM levels are known to control mtDNA copy number. Our results show that most TFAM RNAi flies enter the pharate stage despite having a lower mtDNA copy number than homozygous D263A larvae (Fig. 5e and Supplementary Table 5). This shows that the mtDNA depletion in the homozygous D263A larvae is not sufficient on its own to cause the observed lethality.

### DmPOLγA exo^−^ flies slowly accumulate mtDNA mutations

**Figure 6:**
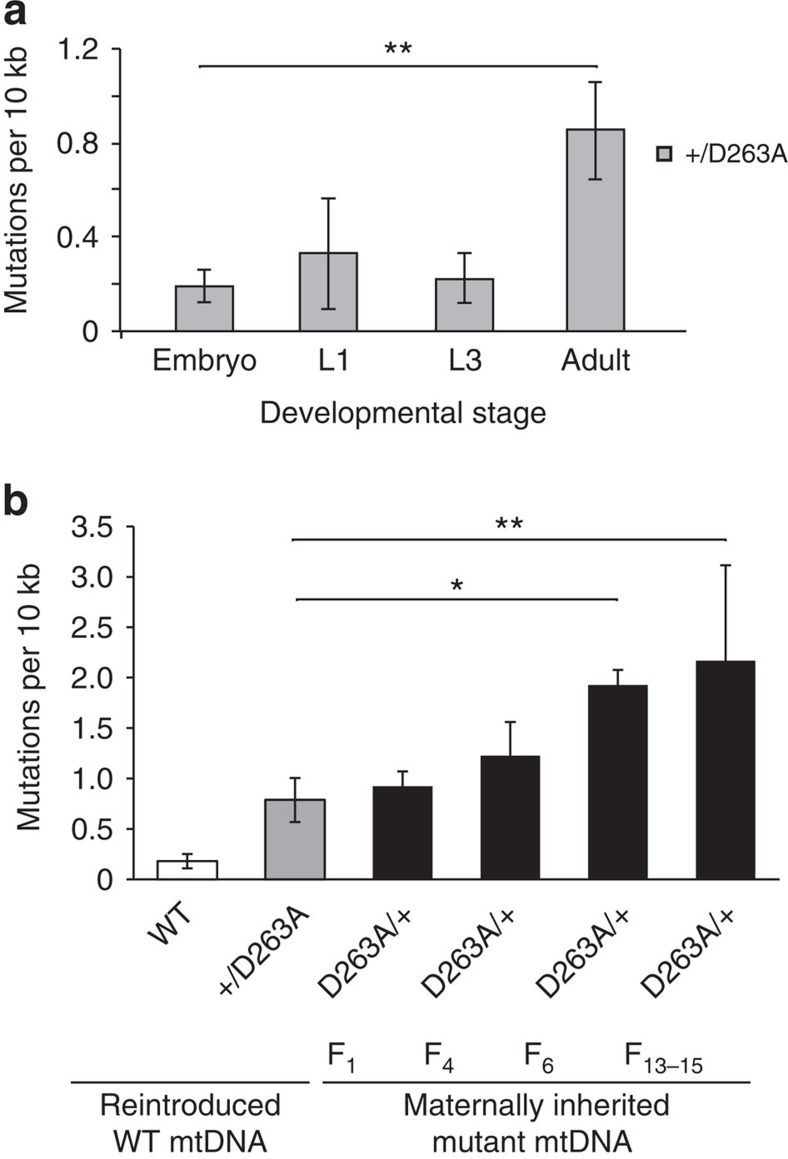
MtDNA mutations increase after morphogenesis and accumulate transgenerationally. (**a**) MtDNA mutation load was determined in heterozygous D263A flies in different developmental stages. The D263A exo^−^ allele was transmitted paternally and therefore all the detected mutations are produced by somatic mutagenesis. An increase in the mtDNA mutation load relative to wild-type (WT) flies was only detected after morphogenesis. One-way ANOVA with Dunnett's *post hoc* test. **P<0.01. Error bars represent s.d. *n*=3–6. (**b**) MtDNA mutations accumulated in heterozygous D263A exo^−^ flies after successive intercrossing for several generations. MtDNA mutation loads were compared among wild-type flies (WT, white bar), heterozygous D263A exo^−^ flies with a clean background (+/D263A, lack maternally transmitted mtDNA mutations, grey bar) and heterozygous D263A exo^−^ flies that maternally inherited mtDNA mutations for 1, 4, 6 or 13–15 generations (D263A/+, black bar). One-way ANOVA with Dunnett's *post hoc* test. **P<0.01, *P<0.05. Error bars represent s.d. *n*=3–6.

We first studied how mutation levels change during development in flies that have only somatic mutagenesis of mtDNA, that is, flies that are heterozygous for a paternally transmitted D263A allele (Fig. 6a). Interestingly, there was a burst in mtDNA mutation levels after morphogenesis (Fig. 6a). Next we proceeded to study how these mtDNA mutations are transmitted between generations. To this end, we first crossed heterozygous D263A male flies to WT females and measured the mtDNA mutation load in the offspring (Fig. 6b). As the analysed flies were born to mothers that only had transmitted WT mtDNA, maternally transmitted mtDNA mutations cannot influence the overall mtDNA mutation levels. This cross thus allowed us to selectively determine the somatic mtDNA mutation load generated by the heterozygous D263A allele (Fig. 6b). Next, we performed crosses with female flies that had inherited their mtDNA from a maternal lineage of flies heterozygous for the D263A allele for 1, 4, 6 or 13–15 generations (Fig. 6b). Surprisingly, we observed that the accumulation of mtDNA mutations between generations occurred very slowly in flies (Fig. 6b). In fact, the mtDNA mutation load in flies heterozygous for the D263A allele was very similar in strains with reintroduced WT mtDNA and in strains intercrossed for up to four generations (Fig. 6b). However, on further intercrossing, for six generations or more, increased mtDNA mutation levels were observed (Fig. 6b) and the total mutation levels were comparable to those of homozygous D263A larvae (Fig. 4). Altogether, these observations show that flies can maintain a wider mtDNA sequence variation between generations than mammals^41^. It is generally accepted that a small genetic bottleneck in the mammalian maternal germline cause rapid shifts in mtDNA genotypes between generations^41^. Therefore, the slow accumulation of mtDNA mutations between generations in heterozygous D263A flies suggests that there is a wider size of the genetic bottleneck for maternal transmission of mtDNA in flies than in mammals.

### The DmPOLγA exo^−^ and pol^−^ variants can complement in trans

**Figure 7:**
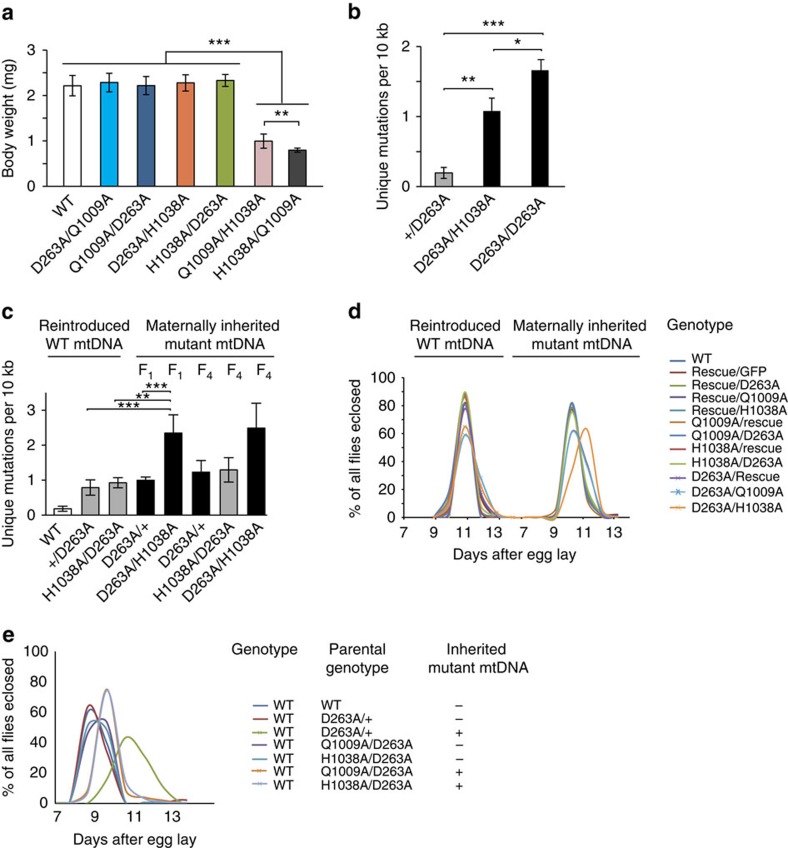
Genetic complementation at the *tamas* locus. (**a**) Comparison of body weight between different *DmPOLγA* compound-heterozygous larvae. All *DmPOLγA* compound heterozygous larvae had wild-type-like body size. Body weight of 20 larvae (5-day old) was measured and average body weight is shown. Tukey's Multiple Comparison Test. ***P<0.001, **P<0.01. Error bars represent s.d. *n*=20. (**b**) Quantification of unique mtDNA mutations in compound heterozygous larvae. The homozygous exo^−^ larvae had significantly more unique mtDNA mutations in comparison with compound heterozygous larvae. Both genotypes inherited mtDNA mutations maternally for one generation. Tukey's Multiple Comparison test. ***P<0.001, **P<0.01, *P<0.05. Error bars represent s.d. *n*=3. (**c**) Quantification of unique mtDNA mutations in adult compound heterozygous flies without and with maternally transmitted mutations. Compound heterozygous flies with maternally transmitted D263A exo^−^ allele (black bar) showed increase in the number of unique mtDNA mutations as compared with compound heterozygous flies that inherited the exo^−^ allele paternally (grey bar). One-way ANOVA with Dunnett's *post hoc* test. ***P<0.001, **P<0.01. Error bars represent s.d. *n*=3–6. (**d**,**e**) An increase in the mtDNA mutation load affects fly development. Developmental time of different complementation groups is shown. (**d**) All genomically engineered flies with a clean background (lacking maternally transmitted mtDNA mutations, left panel) had the same developmental time as wild-type (WT) flies. In contrast, all flies inheriting mtDNA mutations maternally for four generations (D263A/Rescue, D263A/H1038A and D263A/Q1009A) showed developmental delay (right panel). Crossing schemes are shown on Supplementary Fig. 7a. Data represent two independent experiments. (**e**) Wild-type (WT) flies carrying mtDNA mutations showed a severe developmental delay. Compound heterozygous flies used in **d** were outcrossed twice to replace *tamas* mutant alleles with wild-type *tamas* allele and clean the nuclear background. All flies had a WT nuclear background with or without maternally inherited mtDNA mutations.

The finding that flies that are heterozygous for the D263A allele have about half of the mtDNA mutation load of larvae that are homozygous for the D263A allele suggest that the WT allele can complement in trans. We performed a series of crosses to obtain compound heterozygous flies to investigate the occurrence of genetic complementation (Supplementary Fig. 7a). The two pol^−^ mutants Q1009A and H1038A could not complement each other to generate viable compound heterozygous flies (Supplementary Table 2). However, compound heterozygotes, expressing either of the pol^−^ mutants from one allele and the exo^−^ mutant from the other allele, were viable and apparently normal (Fig. 7a and Supplementary Fig. 7b–d and Supplementary Table 2). We observed no differences in body weight (Fig. 7a) or oxygen consumption rates (Supplementary Fig. 7b) of these compound heterozygous larvae in comparison with controls, irrespective of whether the D263A allele was paternally (Q1009A/D263A and H1038A/D263A) or maternally (D263A/Q1009A and D263A/H1038A) inherited. Furthermore, we found no differences in total mtDNA levels between compound heterozygous and WT larvae (Supplementary Fig. 7c) and no linear mtDNAs with deletions were present (Supplementary Fig. 7d). Based on these findings, we conclude that compound heterozygotes harbouring one exo^−^ (D263A) and one pol^−^ allele (Q1009A or H1038A) are phenotypically normal due to genetic complementation.

### Complementing flies have clonally expanded mtDNA mutations

We proceeded to investigate the mtDNA mutation load in larvae to get insights into why compound heterozygous (D263A/H1038A) but not homozygous exo^−^ (D263A/D263A) larvae can proceed normally through development. There is accumulation of mtDNA mutations after morphogenesis (Fig. 6a) and any meaningful comparison of the mutation load must therefore be performed in larvae at the same developmental stage. The D263A/H1038A larvae had a similar total mutation load as the D263A/D263A larvae at the L3 stage (Supplementary Fig. 7e), but the number of unique mtDNA mutations was significantly lower (Fig. 7b). The high unique mtDNA mutation load and the presence of linear mtDNA deletions in D263A/D263A larvae likely explain why they cannot proceed through development.

Next, we studied adult flies that were compound heterozygous or only heterozygous for the D263A allele and found similar mutation loads if the D263A allele was paternally transmitted (Fig. 7c). In contrast, compound heterozygous flies containing a D263A allele that had been maternally transmitted for one generation had a higher mtDNA mutation load (Fig. 7c). We maintained heterozygous D263A mutant flies by intercrosses for four generations and then crossed the obtained D263A females with H1038A males to generate compound heterozygotes (D263A/H1038A) (Supplementary Fig. 7a). Very unexpectedly, we observed a substantial increase in the total mtDNA mutation load in the compound heterozygous adult flies, which is mainly explained by high levels of clonally expanded mutations (Supplementary Fig. 7f), thus showing that the H1038A allele likely decreases the size of the bottleneck in the maternal germline. It should be noted that mtDNA mutations that have passed through the germline are subject to purifying selection^29^,^31^,^39^ and therefore are less deleterious than mtDNA mutations that are somatically generated, which likely explains why the compound heterozygous flies are viable despite having high total mtDNA mutation loads.

### Maternal mtDNA mutations cause developmental delay

We investigated the timing of development in different types of compound heterozygous flies, where the females contained re-introduced WT mtDNA, and in all cases the majority of flies eclosed at ∼11 days AEL (Fig. 7d, left panel). However, the presence of the D263A allele in the mother led to a slight delay of eclosion in a subpopulation of flies (Fig. 7d, left panel), consistent with some germline mutagenesis of the re-introduced mtDNA. Next, we maintained heterozygous D263A mutant flies by intercrosses for four generations and then crossed them to obtain compound heterozygotes (Fig. 7d, right panel). Interestingly, significant developmental delay was observed in the compound heterozygous flies coming from maternal lineages where mtDNA mutations could accumulate (D263A/Rescue, D263A/H1038A and D263A/Q1009A; Fig. 7d, right panel). To exclude the possibility that acquired nuclear mutations could explain the observed developmental delays, we performed outcrosses to WT flies for two generations. Consistent with a maternal effect, we observed significant developmental delay only in the WT progeny with inherited mtDNA mutations (Fig. 7e). In this experiment, we also included heterozygous D263A flies that had been maintained by intercrosses for ∼2 years and outcrossed to WT flies for six generations (Fig. 7e, green and red lines). A very significant developmental delay was observed in the outcrossed flies that were maternally related to the original cross (Fig. 7e, green line), whereas paternally related flies had normal timing of development (Fig. 7e, red line).

### HsPOLγA exo^−^ and pol^−^ proteins cooperatively replicate mtDNA

**Figure 8:**
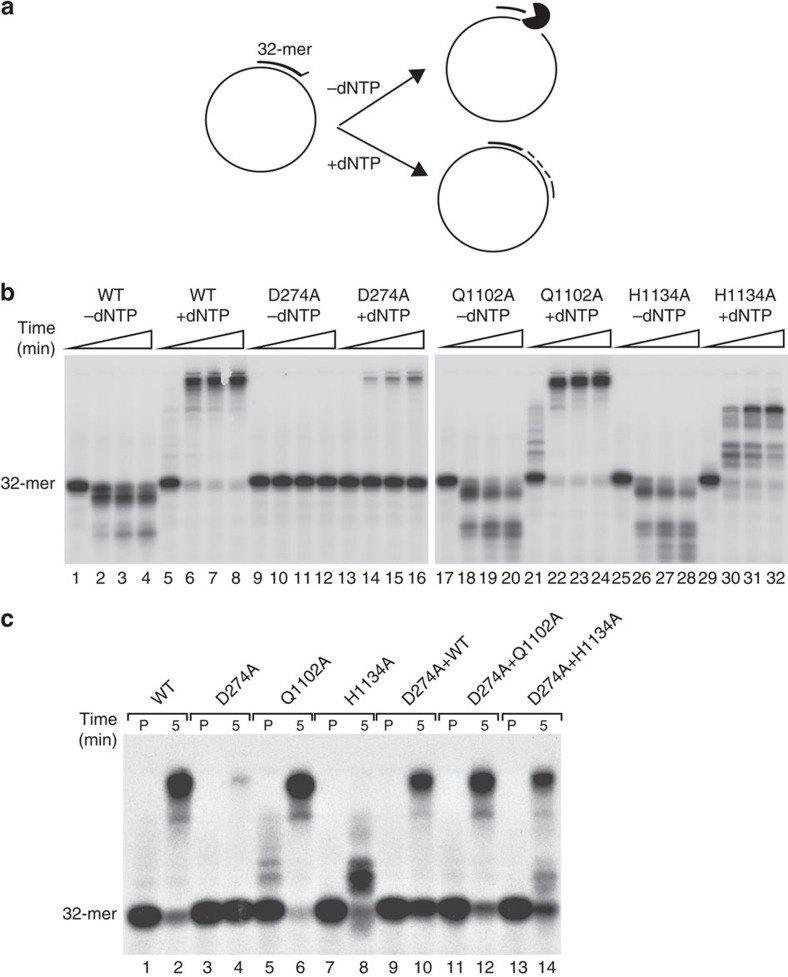
Mutant recombinant HsPOLγA proteins can complement each other in *in vitro* assays. (**a**) Schematic overview of the HsPOLγA *in vitro* proofreading and polymerase activity assays. The template used in these assays consisted of a 5′ ^32^P-labelled 32-mer annealed to single-stranded pBluescript SK(+) creating one-nucleotide mismatch at the 3′-end. In the absence of dNTPs, HsPOLγA will backtrack (3′ to 5′ direction), degrading the oligonucleotide whereas in the presence of dNTPs the polymerase will proofread the mismatch and polymerize from the 3′-end. (**b**) The proofreading-deficient D274A had no exonuclease activity (lane 9–12), which led to impaired ability to continue DNA synthesis from the mismatched primer end (lane 13–16). The Q1102A mutant showed both extensive proofreading (lane 17–20) and polymerase activities (lane 21–24) comparable to the WT enzyme (lane 1–8). The H1134A mutant had extensive exonuclease activity (lane 25–28) but was a poor polymerase only able to copy a short stretch of DNA (lane 29–32). Triangles indicate elapsed time with 15 min being the end point. (**c**) The D274A and H1134A mutants were not able to copy DNA alone from the mismatched primer end (lanes 4 and 8, respectively). However, by mixing the two mutant proteins we observed a synergistic effect (lane 14) suggesting cooperation at the mismatched primer-end. The cooperation of D274A with WT and Q1102A is not as pronounced as in this assay (lane 10 and 12). ‘P' means the samples were pre-incubated on ice for 5 min and then stopped. Reactions labelled ‘5' were also pre-incubated on ice for 5 min but followed by a 5-min incubation at 37 °C before stopping the reaction. All experiments were repeated at least three time.

To investigate whether we could reconstitute the observed genetic complementation biochemically with recombinant mutant forms of HsPOLγA, we assessed elongation of a DNA primer containing a 3′ mismatch (Fig. 8a). The WT HsPOLγA could remove the 3′ mismatch by its exonucleolytic activity and then proceed to elongate in an efficient manner, whereas the D274A mutant could not efficiently remove the mismatch and switch to elongation (Fig. 8b). In contrast, both the Q1102A and H1134A mutants displayed increased exonuclease activity (Fig. 8b) in comparison with WT HsPOLγA (Fig. 8b). The Q1102A mutant was efficient in elongation, whereas the H1134A mutant was inefficient (Fig. 8b), which is in agreement with the severely reduced polymerase activity of the H1134A mutant (Fig. 1e). Finally, we performed *in vitro* complementation assays by mixing the D274A mutant with either Q1102A or H1134A mutants, using a replication primer that carried a 3′ mismatch nucleotide. On their own, neither the D274A nor the H1134A mutant was able to synthesize DNA efficiently (Fig. 8c, lanes 4 and 8). However, when we mixed the two mutant proteins, they complemented each other and supported efficient DNA synthesis (Fig. 8c, lane 14) suggesting cooperation at the mismatched primer end. The exonuclease and polymerase domains are located in separate regions of HsPOLγA and the observed complementation between D274A and H1134A mutants shows that defects in different functional domains of POLγA can complement each other (Fig. 8c, compare lane 4 and 8 with lane 14). To accomplish this, complementation POLγA must frequently go on and off the template during exonucleolytic proofreading and elongation.

## Discussion

Faithful replication has long been assumed essential for mtDNA to prevent the accumulation of deleterious mutations in the absence of recombination. In addition, a yet not fully resolved mechanism during embryogenesis ensures that only a limited number of mtDNA molecules are transmitted through the female germline to the next generation. Flies seem to be more susceptible to impaired mtDNA maintenance than the mutator mice, because the majority of flies homozygous for the exo^−^ DmPOLγA did not pass the L3 larval stage, despite carrying approximately threefold less mtDNA mutations than mtDNA mutator mice^41^,^42^,^43^,^53^. Similar to the situation in mtDNA mutator mice, the majority of mtDNA mutations accumulated during early developmental stages also in flies. The finding of arrested development, caused by high levels of mtDNA mutations or mtDNA depletion, show that mitochondrial function is important to allow larvae to proceed to the pupal stage. Interestingly, heterozygous exo^−^ flies have lower mtDNA mutation levels than homozygous exo^−^ larva and no obvious phenotype. These findings argue that mtDNA mutations will only impair development if the mtDNA mutation levels exceed a critical threshold. The mutational pattern observed in the exo^−^ flies is analogous to the pattern in mtDNA mutator mice and consists mainly of transition mutations. The majority of mtDNA mutations are transitions also in wild animals and humans, which is consistent with the hypothesis that most of the mtDNA variation is created by replicative errors rather than by unrepaired base lesions caused by oxidation or other types of damage^22^,^24^.

In accordance with results from mtDNA mutator mice^22^,^42^, homozygous exo^−^ larvae contained linear mtDNA molecules with deletions. In mice, the linear deleted mtDNA molecule spans the major arc between both origins of replication, and the levels of deleted mtDNA does not increase with time, which suggests they are formed by abortive replication of the full-length mtDNA molecule and are not themselves templates for mtDNA replication. We have recently demonstrated that the exonuclease activity of mammalian POLγ is required to create ligatable ends at the termination of replication^54^. Inactivation of the exonuclease activity causes the appearance of unligated nicks at the origins of replication, which leads to linear deletions during subsequent rounds of mtDNA synthesis. The linear deletion observed in mouse therefore defines the location of the strand-specific origins for mtDNA synthesis^54^. In flies, the origins of mtDNA replication have not yet been defined, although the noncoding A+T-rich control region has been proposed to contain both origins in arthropods^52^. In the homozygous exo^−^ larvae, we mapped the ends of the linear deletions to the A+T-rich region. Our observation of linear deletions and the location of their ends therefore strongly suggests that also fly mtDNA, similar to mammalian mtDNA, has a dedicated origin of replication for each strand of mtDNA, and that these origins are located in the non-coding A+T-rich region of fly mtDNA.

Further, our results suggest that flies have a wider bottleneck during transmission of mtDNA through the maternal germline than mammals^29^. In mice, continuous breeding maternal lineages of heterozygous mtDNA mutator mice results in rapid accumulation of mtDNA mutations within a few generations^29^, whereas similar crosses of heterozygous exo^−^ flies required six generations before an additional increase of the mtDNA mutation load could be observed. Interestingly, we observed that compound heterozygous flies showed a much more rapid clonal expansion of mtDNA mutations than exo^−^ heterozygous flies. This is likely due to a post-fertilization bottleneck as the polymerase-deficient high-fidelity allele may reduce mtDNA copy number. Similar observations were made in the mouse, where mtDNA copy number during early development is reduced in heterozygous TFAM KO mice^49^. It is thus tempting to suggest that while the mammalian mitochondrial bottleneck results in low variation, flies transmit a larger number of mtDNA copies^55^ and thus tolerate more somatic mtDNA variation. The short lifespan of flies may thus not have led to the selection of a tight bottleneck during evolution.

Surprisingly, our results from the complementation studies suggest that the mtDNA polymerase can go on and off the template during replication and that, despite being incapable of replicating mtDNA in an efficient manner, the pol^−^ variants may have been able to repair some of the replication errors introduced by the exo^−^ mutant. This observation could very well explain the molecular nature behind disease-causing dominant versus recessive mutations in the *POLγA* gene in humans. Several autosomal recessive mutations have been described and one of the most common is the A467T mutation, which has been found alone or *in trans* with other mutations in the same gene^56^. *In vitro* studies suggest that only 4% of the polymerase activity remains in A467T mutant alleles^57^. Heterozygous carriers are healthy, showing that the WT allele can complement the defect. In contrast, autosomal dominant mutations in the *POLγA* such as the Y955C leading to progressive external ophthalmoplegia (adPEO) stalls mtDNA replication at specific sites *in vitro*^58^. Autosomal dominant mutations thus might compete with the WT allele preventing full replication, and thereby cause mtDNA deletions or depletion.

In conclusion, we show here that mtDNA mutations transmitted through the fly germline are clonally expanded and they will severely impact fly development when they are present above a certain threshold. Furthermore, we provide genetic evidence that two *PolγA* alleles, mutated in different domains, can functionally complement each other *in vivo* and *in vitro*, suggesting that mtDNA polymerase can re-initiate mtDNA replication after an aborted replicative cycle. Finally, our data suggest that the exonuclease activity of POLγA is required for mtDNA replication to proceed past the origins of replication.

## Methods

### Expression and purification of recombinant human proteins

Mutagenesis of the human POLγA variants was carried out using the QuickChange Lightning Site-directed mutagenesis kit according to the provided protocol (Agilent). The presence of HsPOLγA mutations was confirmed by sequencing (Eurofins MWG Operon). Protein expression and purification of the different POLγA variants, POLγB, TWINKLE and mtSSB were done as previously described^8^. The concentrations of the HsPOLγA mutants were determined by quantification from SDS–PAGE using WT HsPOLγA as reference.

### EMSA and coupled exonuclease/polymerase assays

DNA affinity of POLγ to a primer template was assayed using EMSA^59^. The reactions contained 10 fmol DNA template and the indicated holoenzyme (POLγA/POLγB complex) concentrations in the presence of 300 μM ddGTP and 3 mM dCTP. The reactions were run on 6% native polyacrylamide gels in 0.5 × TBE for 35 min at 180 V and visualized by autoradiography. Each experiment was repeated three times. Band intensities representing unbound and bound DNA were quantified using Fujifilm Multi Gauge V3.1 software. The fraction of bound DNA was determined from the background-subtracted signal intensities using the expression: bound/(bound+unbound). The fraction of DNA bound in each reaction was plotted versus the concentration of POLγ. Data were fit with the binding equation (Fraction bound=(MaxB × [POLγ])/(MaxB+[POLγ]) using EXCELs Add-in ‘Solver' to perform non-linear regression and obtain values for Kd (as the value corresponding to the midpoint of MaxB) and using MaxB set to 1 (the fraction bound at which the data plateaus). The EMSA substrate was also utilized to examine the polymerization and the 3′ to 5′ exonuclease activities of POLγA as previously described^60^ but with 150 fmol of the indicated POLγA variant and 600 fmol of POLγB. The reactions were analysed on a 15% denaturing polyacrylamide gel electrophoresis in 1 × TBE and visualized by autoradiography.

### Second strand DNA synthesis

A ^32^P-labelled 32-mer (5′- CTATCTCAGCGATCTGTCTATTTCGTTCATCC -3′) was annealed to SS pBluescript SK(+). DNA synthesis reactions were performed as described previously and were incubated at 37 °C for the times indicated^60^. The products were analysed by electrophoresis in a 0.9% agarose gel and visualized by autoradiography.

### *In vitro* rolling circle DNA replication

An oligonucleotide consisting of 70 nucleotides (5′-42[T]- ATCTCAGCGATCTGTCTATTTCGTTCAT -3′) was hybridized to a SS pBluescript SK(+) followed by one cycle of polymerization with KOD polymerase (Novagen) to produce an ∼3-kb double-stranded template with a preformed replication fork. The reactions were performed as described^61^, and were incubated at 37 °C for times indicated (0, 5, 15, 30 and 60 min). The products were analysed by electrophoresis in a 0.8% denaturing agarose gel and visualized by autoradiography.

### 3′–5′ exonuclease activity

A 5′ ^32^P-labelled 32-mer (5′- CTATCTCAGCGATCTGTCTATTTCGTTCATCG -3′) with a one-nucleotide mismatch at the 3′-end was annealed to SS pBluescript SK(+). The reactions were performed as in the second strand DNA synthesis assay but in the absence or presence of dNTPs and with indicated POLγA variant. The reactions were incubated at 37 °C for the times indicated. The samples were analysed by electrophoresis in a 20% denaturing PAGE and visualized by autoradiography.

### Processivity assays

Assays were performed by mixing 10 μl of reaction mixture A with 10 μl of reaction mixture B followed by incubation at 37 °C for 10 min. Reaction mixture A contained 25 fmol template as described in Fig. 1d for second strand synthesis, 50 fmol of the indicated POLγA versions and 200 fmol of POLγB, 1 mM DTT, 25 mM TrisHCl pH 7.8 and 0.1 mg ml^−1^ BSA. Reaction mixture B contained 10 mM MgCl_2_, 100 μM dNTP and 0,001 mg ml^−1^ heparin (heparin was omitted in reactions that allowed for multiple binding events to occur). Reactions were stopped by the addition of 20 μl of 95% formamide, 20 mM EDTA and 0.1% bromophenol blue. The samples were analysed by electrophoresis on 12% denaturing PAGE.

### Drosophila stocks and maintenance

Deficiency lines (Df(2L)Exel7059, Df(2L)BSC252, Df(2L)BSC694) and hypomorphic tamas alleles tam3 and tam4 were obtained from Bloomington stock center. TFAM knockdown lines (#1: ID107191 and #2: ID37819) were purchased from the Vienna Drosophila RNAi center (VDRC, Austria). All genomically engineered fly strains were constantly backcrossed into a white Dahomey Wolbachia-free (wDahT) WT strain to avoid accumulation of mtDNA mutations unless stated otherwise. All fly stocks were maintained at 25 °C on a 12:12 h light/dark cycle with 65% humidity and fed on a sugar/yeast/agar (SYA) diet^62^.

### Generation of genomically engineered DmPOLγA flies

Experimental flies were generated by genomic engineering in a two-step process^48^. In the first step, we generated a DmPOLγA KO founder line by replacing the endogenous DmPOLγA gene (tamas) with an attP site by ends-out homologous recombination. The attP site was then used in the second step to reintroduce wild-type and mutated versions of the tamas gene by Φ31-mediated recombination.

For the ends-out homologous recombination donor construct ∼4 kb of 5′ and 3′ flanking sequences of the tamas gene were cloned into the pBlueScript II SK(+) vector (Stratagene) by ET recombination using gene-specific primers (Supplementary Table 6) and a tamas BAC clone (RP98–30I21, BACPAC Resource Center, Oakland, California). 5′ and 3′ homologous arms were sequence verified and subsequently cloned into the pGXattP targeting vector^48^. Transgenic flies carrying the tamas pGXattP targeting vector were generated by P-element-mediated germ line transformation using the BestGene Drosophila embryo injection service. Crosses for ends-out homologous recombination were carried out according to the rapid targeting scheme^63^. Subsequently, the white^hs^ marker gene was genetically mapped and homologous recombination events were identified by PCR using primers PCR3 and PCR4 (Supplementary Table 6). Tamas KO flies were crossed to flies expressing a cre-recombinase to remove the *white*^hs^ marker gene^48^. Absence of the tamas gene was confirmed by PCR using primers PCR1 (Supplementary Table 6). The corresponding flies are referred to as tamas KO flies.

Rescue construct (Supplementary Fig. 3a) was amplified by PCR using primers PCR8 (Supplementary Table 6). Rescue construct was sequence verified and subsequently cloned using XhoI and AscI sites into the pGEattBGMR vector^48^. Mutagenesis of the WT tamas allele was carried out using the ‘QuickChange Lightning Site-directed mutagenesis kit' according to the provided protocol and pGEattBGMR-Rescue construct was used as a template. Primers used for site-directed mutagenesis are indicated in Supplementary Table 6. All mutant tamas allelic variants were sequenced to ensure the absence of base substitutions. The presence of D263A, H1038A and/or Q1009A mutations was confirmed by sequencing. Mutant variants of the tamas gene were then injected into the embryos of tamas KO flies expressing Φ31 integrase by the in-house *Drosophila* transgenic core facility. Primers used to verify the precise re-introduction of tamas allelic variants into the native tamas locus were PCR5 and PCR6 (Supplementary Table 6). The absence of genomic rearrangements on re-introduction of tamas mutant alleles was confirmed by Southern blot analyses. Total DNA was isolated from the corresponding genotypes and digested with XhoI. A full-length complementary DNA (cDNA) of DmPOLγA was used as a probe and the signal was visualized by autoradiography.

To verify that the white^hs^ marker gene did not interfere with DmPOLγA function and induced lethality, all tamas mutant lines were crossed to flies expressing the cre-recombinase. Precise removal of the white^hs^ marker gene was confirmed by PCR using primers PCR5 and PCR7 (Supplementary Table 6). Tamas mutant flies with or without the white^hs^ marker gene showed similar survival, thus for all experiments tamas mutant flies carrying the white^hs^ marker gene were used.

### Fly developmental time

To assess developmental time, flies were allowed to lay eggs on grape juice agar plates for 3 h. For each genotype, 100 eggs were individually picked and placed into vials with SYA food. The number of eclosed flies was scored every 12 h. At least five biological replicates were done for each genotype.

### Developmental analysis of DmPOLγA mutant flies

For assessing the percentage of DmPOLγA mutant flies entering L3 stage, flies were allowed to lay eggs on grape juice agar plates for 2 h. For each genotype, 100 eggs were individually picked and placed into vials with SYA food. The number of L3 larvae of each genotype was scored 6 days AEL. At least six biological replicates were done for each genotype.

To assess the percentage of DmPOLγA mutant flies entering the pupal stage, 50 L3 larvae were collected and transferred into vial with SYA food. The number of pupae was scored every 12 h. At least three biological replicates were done for each genotype.

To assess the percentage of DmPOLγA mutant flies entering the adult stage, 50 L3 larvae were collected and transferred into vial with SYA food. The number of adult flies was scored every 12 h. At least three biological replicates were done for each genotype.

### DNA isolation and qRT–PCR and Southern blot analyses

For relative mtDNA copy number determination, total DNA extractions were prepared from L3 larvae using DNeasy Blood and Tissue Kit (Qiagen). Five biological replicates, each with 10 larvae, were prepared for each genotype. Quantification of mtDNA levels was done using SYBR-Green qPCR analyses and primers targeting cytB and rpL32 (Supplementary Table 6). All data were normalized against wild type levels.

For Southern blot analysis, total DNA was extracted from 20–30 L3 larvae. Samples were homogenized with a tissue grinder in 400 μl of buffer A (100 mM Tris-HCl, pH 7.5; 100 Mm EDTA; 100 mM NaCl and 0.5% SDS). After incubation at 65 °C for 30 min, 800 μl of Buffer B (4 ml of 5 M KOAc and 10 ml of 6 M lithium chloride) was added and samples were left on ice for 60 min. After incubation, samples were centrifuged at 12,000*g* for 15 min and supernatant was transferred into a new tube. About 540 μl of isopropanol was added to the supernatant and samples were further centrifuged at 12000*g* for 15 min. Pellet was washed with 70% ethanol, dried and resuspended in 100 μl nuclease-free water containing 20 μg ml^−1^ RNase A. After incubation at 37 °C for 1 h, samples were stored at +4 °C.

Approximately, 1–3 μg of total DNA was cut using either EcoRV, PstI, NdeI, NsiI or StyI restriction endonuclease. Digestions were run on 0.8% agarose gels, and blotted to Hybond-N+ membrane (Amersham Bioscience). ND2, COXI or 12S rRNA were used as probes and signals were visualized by autoradiography. ^32^P-labelling of Southern probes was done according to manufacturer's instructions (Prime-IT II Random Prime Labeling Kit, Agilent). Primers used to prepare the mtDNA probes can be found in the Supplementary Table 6.

### RNA isolation and quantitative RT–PCR

Total RNA was extracted from 5-day-old larvae using ToTALLY RNA isolation kit (Ambion). For expression analyses, RNA was DNase treated using Turbo DNA-free kit (Ambion) and reverse transcribed using High capacity cDNA Reverse Transcription kit (Applied Biosystems). The expression profiles were determined by quantitative reverse transcription–PCR (qRT–PCR) analyses with a 7900HT Fast Real Time PCR System (Applied Biosystems). Rpl32 was used as a normalization control^64^. Taqman probes were provided by Applied Biosystems under following assay numbers: tamas (Dm01841857_g1); CG8978 (Dm01807408_g1); CG7833 (Dm01842615_g1); CG7811 (Dm01841741_g1); CG33650 (assay is not available anymore).

### MtDNA mutation load analysis

Total DNA was extracted from whole larvae or from the thorax of adult male flies using the DNeasy Blood and Tissue Kit. The mtDNA mutation load was determined by PCR, cloning and sequencing as described elsewhere^22^,^42^,^65^. Fly mtDNA-specific primers were used to amplify a 1.2-kb region encompassing Leu-transfer RNA and parts of COXI and COXII (nucleotide pair 2,194–3,382). An average number of 90 colonies were analysed per animal.

### Biochemical evaluation of respiratory chain function

Five-day-old larvae were collected and the respiratory rates were measured as described by ref. 66. Briefly, 5 L3 larvae were dissected in 100 μl of respiration buffer (120 mM sucrose, 50 mM KCl, 20 mM Tris-HCl, 4 mM KH_2_PO_4_, 2 mM MgCl_2_, 1 mM EGTA, 0,01% digitonin, pH 7.2) and oxygen consumption was monitored at 27 °C using oxygraph (OROBOROS). State 3 respiration was assessed by adding the following substrates: proline (10 mM), pyruvate (10 mM), malate (5 mM), glutamate (5 mM) and ADP (1 mM). State 4 respiration was assessed by adding oligomycin (250 ng ml^−1^) and uncoupled state was obtained by adding 1 μM CCCP. Oxygen consumption rates were normalized to total protein content quantified by the Bradford method (Sigma).

### Statistical analysis

Statistical analyses were performed using GraphPad Prism 5.03 (GraphPad Prism Software, Inc). For statistical analyses of qPCR analyses, qRT–PCR analyses and mtDNA mutation loads one-way ANOVA with Dunnett's test *post hoc* was used unless stated otherwise. For statistical analyses of OXPHOS function Mann–Whitney two-tailed test was used. For statistical analyses of body weight, Tukey's multiple comparison test was used. All data are presented as mean±s.d.

## Additional information

**How to cite this article:** Bratic, A. *et al*. Complementation between polymerase- and exonuclease-deficient mitochondrial DNA polymerase mutants in genomically engineered flies. *Nat. Commun.* 6:8808 doi: 10.1038/ncomms9808 (2015).

## Supplementary Material

Supplementary Information Supplementary Figures 1-7 and Supplementary Tables 1-6.

## Figures and Tables

**Table 1:** The Kd (DNA) values of WT and mutant HsPOLγ holoenzymes (HsPOLγA/HsPOLγB in complex).

**Polγ holoenzyme**	**DNA binding Kd (nM)**
WT	5.5±1.4
D274A	2.8±0.1
Q1102A	3.8±0.2
H1134A	3.5±0.5

WT, wild type.

Table shows values of dissociation constants as an average from three independent binding assays with errors presented as s.d.

**Table 2:** MtDNA mutation pattern in flies carrying the D263A exo^−^ allele.

**Mutation**	**Fraction**		
*Transition*			
A>G	0.13	0.25	0.51
T>C	0.12		
C>T	0.15	0.26	
G>A	0.12		
			
*Transversion*			
A>C	0.00	0.02	0.25
T>G	0.02		
A>T	0.04	0.18	
T>A	0.14		
C>A	0.01	0.05	
G>T	0.04		
C>G	0.00	0.00	
G>C	0.00		
Indel		0.24	

Data were pooled from a total number of 18 flies. In the first column different mutation frequencies are shown from the perspective of the heavy strand. In our method it is not possible to distinguish if the mutation has originally taken place in the heavy strand (that is, AG) or in the light strand (TC). Therefore the frequencies of these indistinguishable mutations have been combined in the second column. In the third column, the frequencies of transitions, transversions and indel mutations are compared showing that most of the detected mutations are transitions (51%).
