# Supplementary material for: Complementation between polymerase- and exonuclease-deficient mitochondrial DNA polymerase mutants in genomically engineered flies
Source: Nat Commun. 2015 Nov 10;6:8808. doi: 10.1038/ncomms9808 (PMC4773887; doi:10.1038/ncomms9808)
Supplement: Supplementary Information — Supplementary Figures 1-7 and Supplementary Tables 1-6. [file ncomms9808-s1.pdf]

SUPPLEMENTARY FIGURES

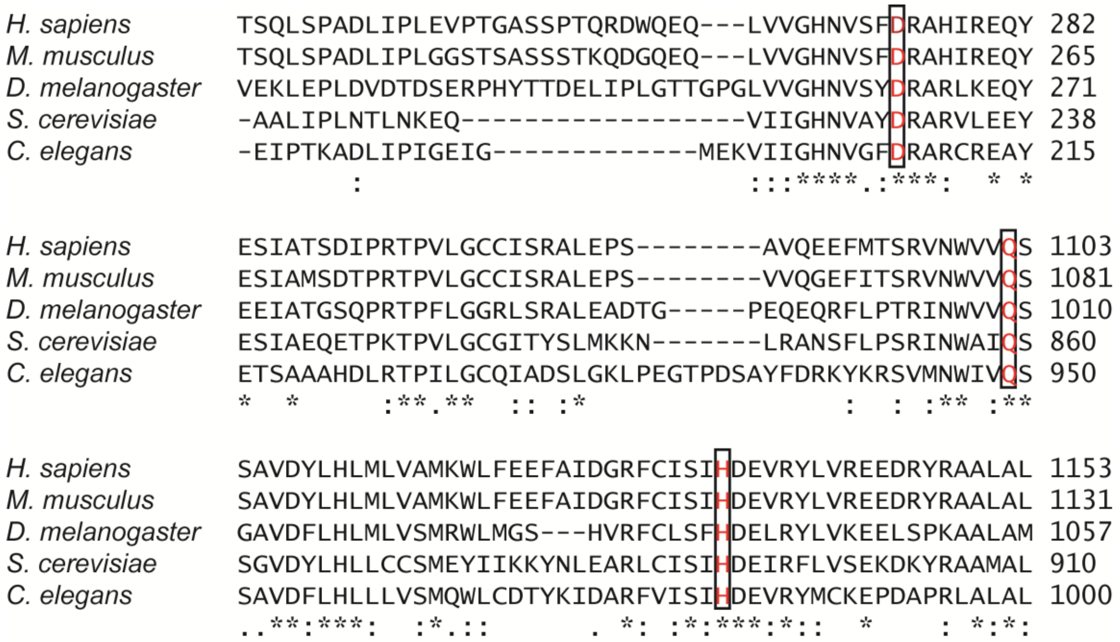

Supplementary Figure 1. Alignment of POLγA protein sequences

Alignment of POLγA protein sequences shows high conservation of the modified POLγA residues among eukaryotes. Conserved amino acids whose codons were altered by genomic engineering in flies (DmD263A, DmQ1009A, DmH1038A) are indicated by boxes. The corresponding amino acids of the recombinant human proteins (HsD274A, HsQ1102A, HsH1134A) are also within boxes. *C. elegans* NP_496592.1, *D. melanogaster* NP_476821.1, *H. sapiens* NP_002684.1, *M. musculus* NP_059490.2, *S. cerevisiae* NP_014975.2.

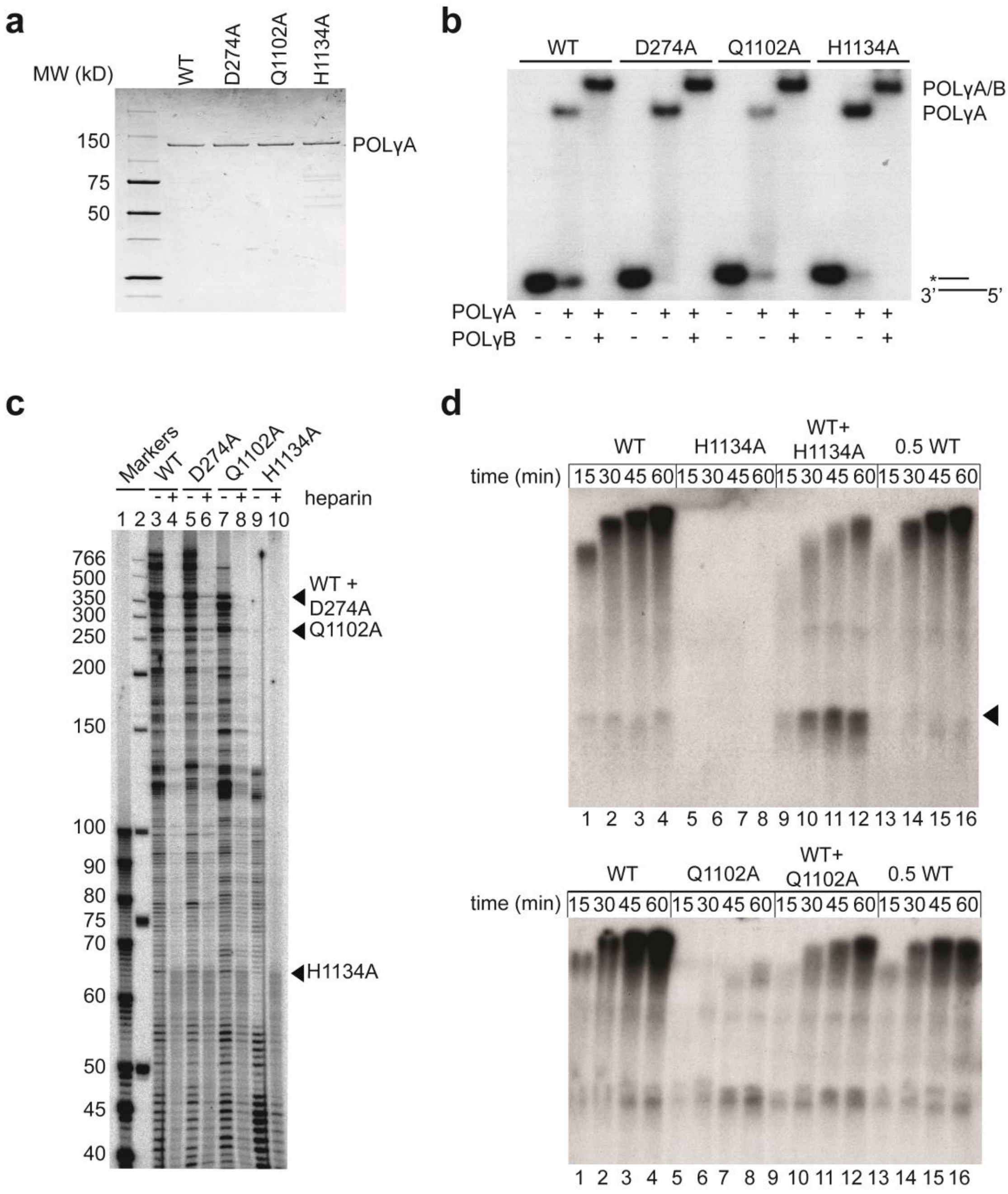

Supplementary Figure 2. Biochemical characterization of the recombinant human POLγA mutants.

(**a**) Coomassie stained 4-20% SDS-PAGE showing purified HsPOLγA proteins of ~140 kDa.

(**b**) EMSA showed that all HsPOLγA proteins bind DNA. Upon addition of HsPOLγB a super-shift is produced showing an interaction also with HsPOLγB.

(**c**) Processivity assays. Processivity assays show that WT (lanes 3-4) and D274A HsPOLγA (lanes 5-6) are processive whereas the Q1102A (lanes 7-8) and H1134A HsPOLγA enzymes (lanes 9-10) are less processive. The H1134A mutant is less processive than the Q1102A.

(**d**) *In vitro* competition assay for the WT and pol- mutant HsPOLγA enzymes. In rolling-circle replication assays, the H1134A HsPOLγA enzyme showed a mild dominant negative effect over the WT enzyme whereas the Q1102A HsPOLγA enzyme did not inhibit the replication of the WT polymerase. The template utilized for rolling circle DNA synthesis is indicated with an arrow-head. In lanes WT, H1134A 150 fmol of enzyme were used. In lanes WT+H1134A 75 fmol of the wild-type and 75 fmol of the mutant enzyme were used. In lane 0.5WT 75 fmol of the wild-type enzyme was used.

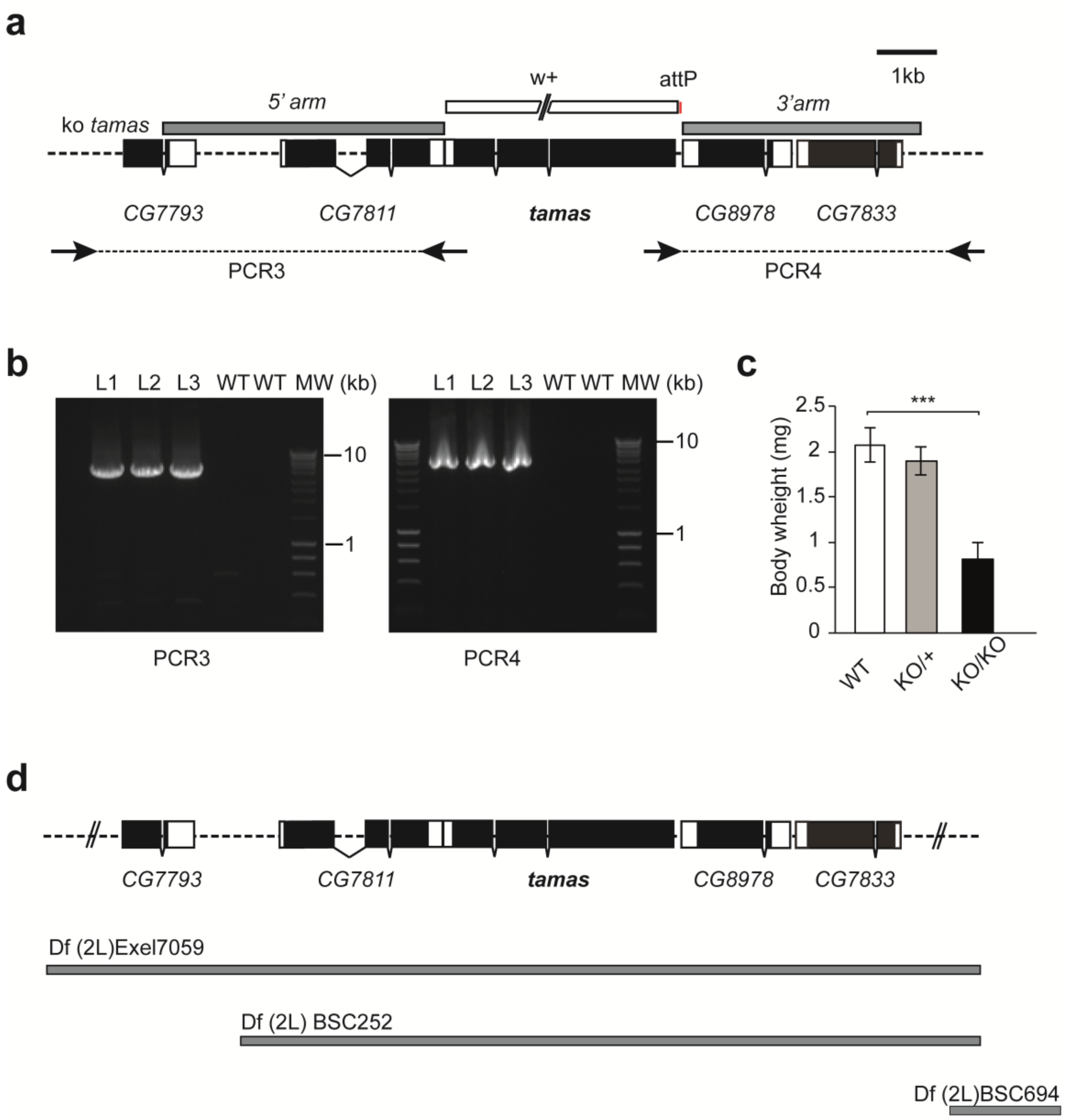

Supplementary Figure 3. Genomic engineering to generate the *DmPOLγA* knockout founder line.

(**a**) The *tamas* locus with adjacent genes and position of the primers used for genotyping are indicated. The donor construct (*ko tamas*) used for ends-out homologous recombination is indicated by grey boxes, while coding sequences are indicated by black and non-translated-regions by white boxes. (**b**) The *DmPOLγA* knockout founder fly line was generated by ends-out homologous recombination. Homologous recombination events were identified by PCR using primers PCR3 and PCR4. Total DNA was extracted

from three independent samples L1-L3, and wild-type (WT) flies were used as control.

(**c**) Comparison of body size between wild-type (WT, white bar), heterozygous (KO/+, grey bar) and homozygous *DmPOLγA* knockout (KO/KO, black bar) larvae. Homozygous knockout larvae were significantly smaller than the wild-type and heterozygous knockout larvae. Data represent at least three independent experiments. One-way ANOVA with Dunnett's post hoc test. ***p<0.001, ** p<0.01, *p<0.05. Error bars represent S.D. n=20.

(**d**) Genetic complementation assay between *DmPOLγA* knockout founder line and deficiency lines. Genetic complementation tests were performed between *DmPOLγA* (*tamas)* knockout line (KO) and deficiency lines that cover (Exel7059, BSC252) or are adjacent (BSC694) to the *tamas* locus.

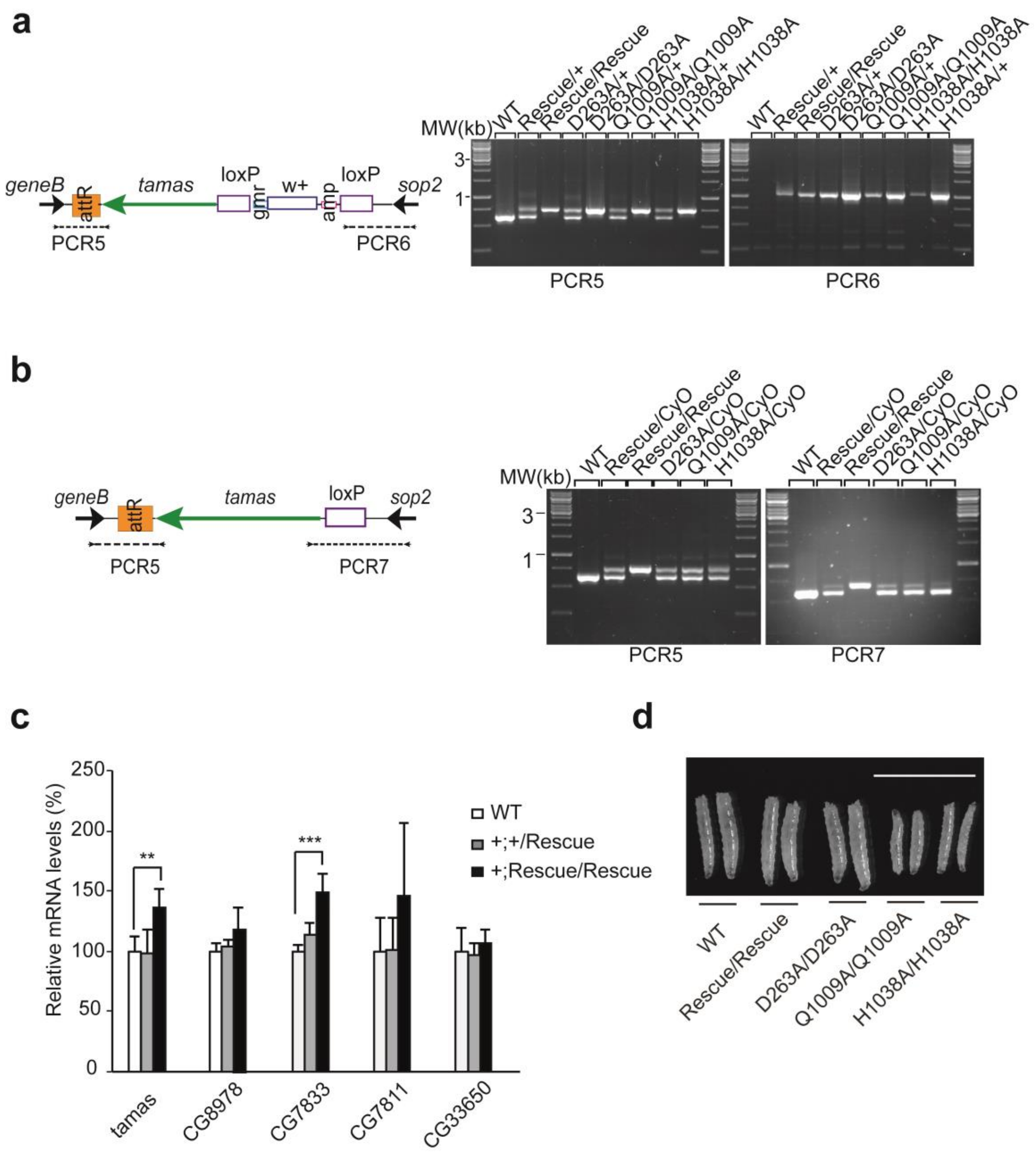

Supplementary Figure 4. Genomic engineering to generate *DmPOLγA* mutant flies

(**a**) PCR verification of the precise integration of *DmPOLγA* mutant alleles into the endogenous *DmPOLγA (tamas)* locus. Schematic depiction of the *tamas* locus after reintegration of the *DmPOLγA* mutant alleles is shown in the left panel. Precise insertion of *DmPOLγA* mutant alleles was confirmed by PCR (right panel). Total DNA was extracted from homozygous and/or heterozygous

genomically engineered rescue, D263A, H1038A and Q1009A *DmPOLγA* flies.

(**b**) Verification of the precise re-integration of *DmPOLγA* variants into the endogenous *tamas* locus after removal of the w+ marker. Schematic depiction of the new *tamas* locus after the reintegration of *DmPOLγA* mutant alleles is shown in the left panel. Precise re-insertion of *DmPOLγA* mutant alleles was confirmed by PCR (right panel).

(**c**) *DmPOLγA* mRNA expression levels and expression of the flanking genes in the *DmPOLγA* rescue flies. Gene expression was analyzed by qRT-PCR and RNA was extracted from 5-day-old larvae and/or adult flies. CG8978/*sop2*, CG7833/*orc5*, CG7811/*b*, CG33650/*DNApol-γ35*. One-way ANOVA with Dunnett's post hoc test. ***$p<0.001$, **$p<0.01$, *$p<0.05$. Error bars represent S.D. n=5.

(**d**) Comparison of body size among genomically engineered *DmPOLγA* larvae. Homozygous DmPOLγA Q1009A and H1038A larvae were substantially smaller than the wild-type (WT), rescue and D263A larvae. Scale bar=5mm.

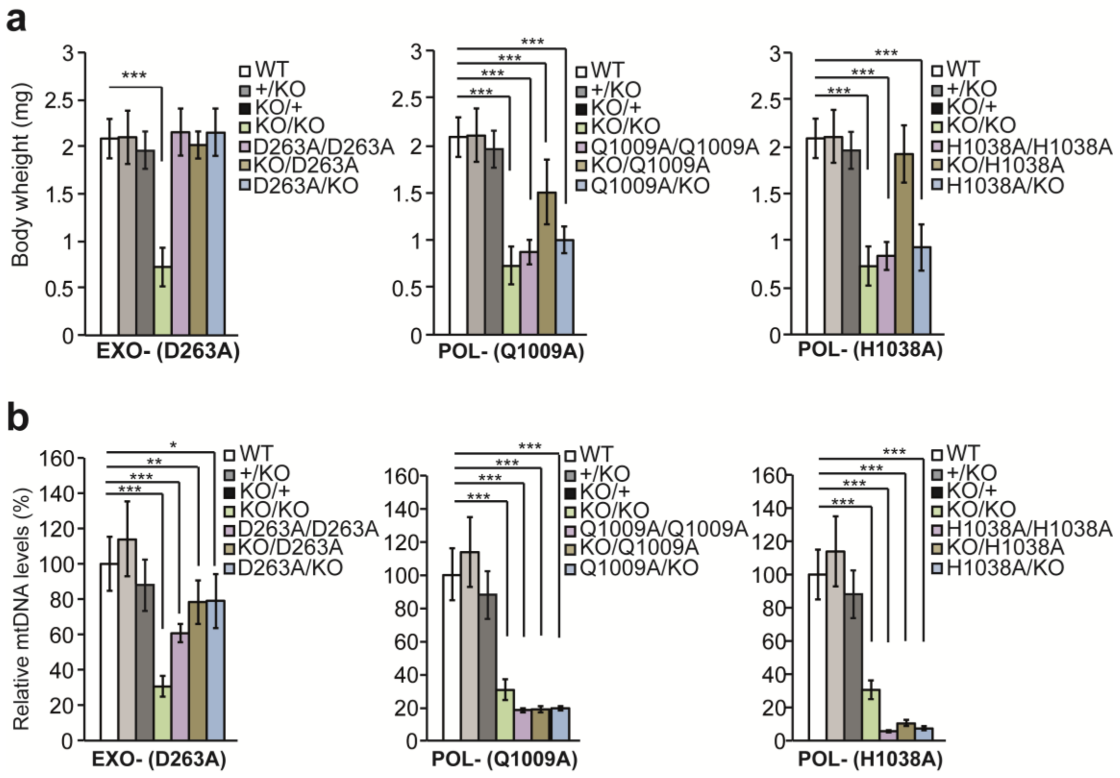

Supplementary Figure 5. Genetic complementation assay between *DmPOLγA* mutant alleles and the *DmPOLγA* KO allele.

(**a**) Quantification of body weight of homozygous and hemizygous DmPOLγA mutant larvae. Hemizygous larvae had almost WT-like body weight but only when the pol- allele was transmitted paternally. One-way ANOVA with Dunnett's post hoc test. ***p<0.001, ** p<0.01, *p<0.05. Error bars represent S.D. n=20.

(**b**) Quantification of relative mtDNA levels of homozygous and hemizygous DmPOLγA mutant larvae. The H1038A allele caused stronger mtDNA depletion when compared to *DmPOLγA* knockout flies (KO) probably due to a dominant negative effect. n=5. In (a) and (b) hemizygous DmPOLγA mutant flies carry one *DmPOLγA* mutant and one *DmPOLγA* knockout allele. In (a) and (b) genotypes are indicated as: maternal allele / paternal allele. All data presented in (a) and (b) are representative of a single experiment that was

carried out three independent times with consistent outcomes. The genotypes were analyzed relative to the WT control within the individual experiment. One-way ANOVA with Dunnett's post hoc test. ***p<0.001, **p<0.01, *p<0.05. Error bars represent S.D.

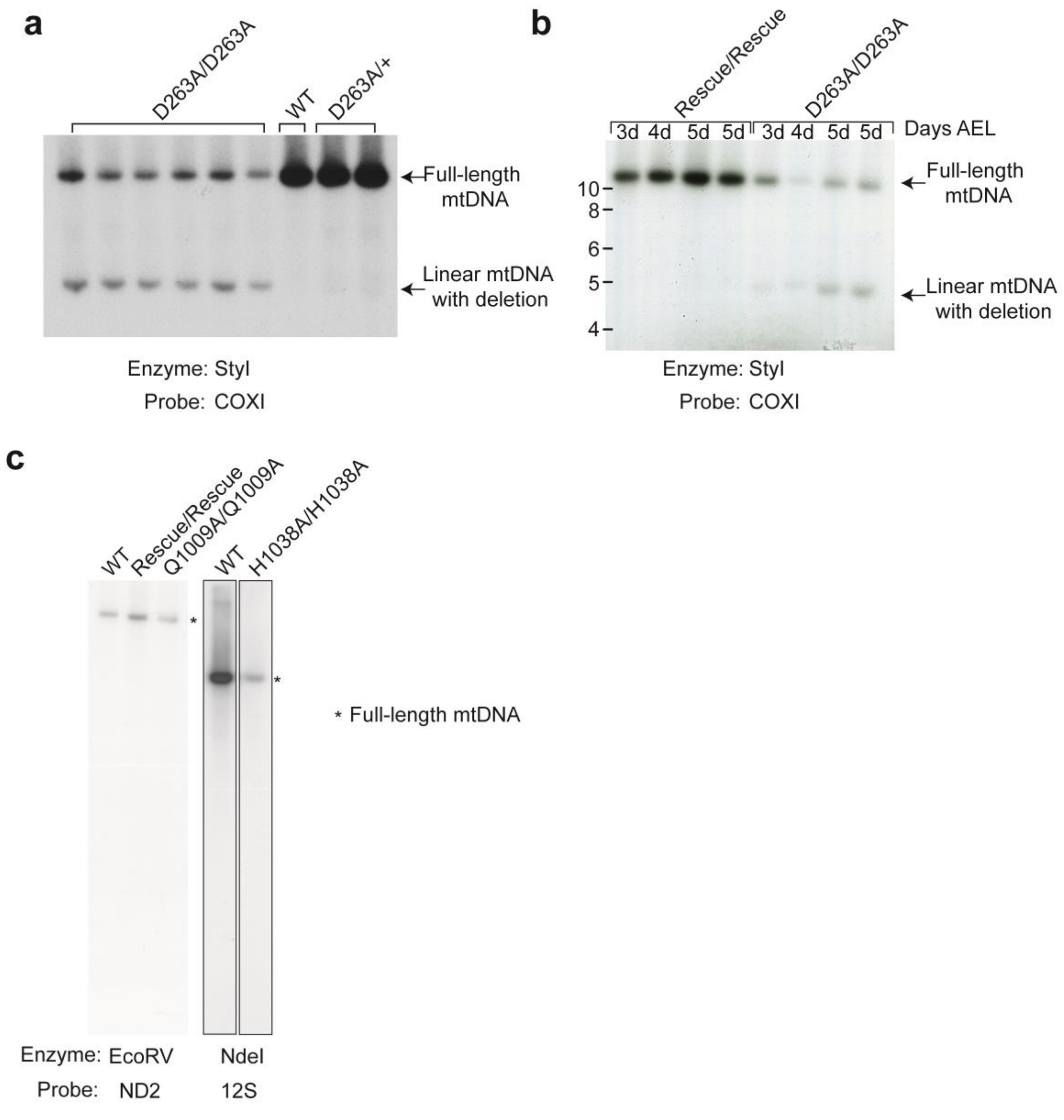

Supplementary Figure 6. Analyses of linear mtDNA fragments in exo- larvae.

(**a**) Formation of linear mtDNA molecules with deletions in homozygous D263A larvae. Linear mtDNA with deletions were detected by Southern blot analyses. Total DNA extracted from 5-day-old larvae was digested with StyI and hybridized with COXI.

(**b**) Linear deleted mtDNA molecules were present already in the 3-day-old D263A larvae. Quantification of levels of mtDNA linear deletions throughout development is shown at the Figure 5d. Total DNA extracted from 3, 4 and 5-day-old larvae was digested with StyI and hybridizied with COXI.

(**c**) Linear deleted mtDNA is not present in H1038A and Q1009A larvae. Southern blot analyses were used to detect the linear mtDNA deletions. Total DNA was extracted from 5-day-old larvae was digested with EcoRV or NdeI and hybridized with ND2 and 12S, respectively.

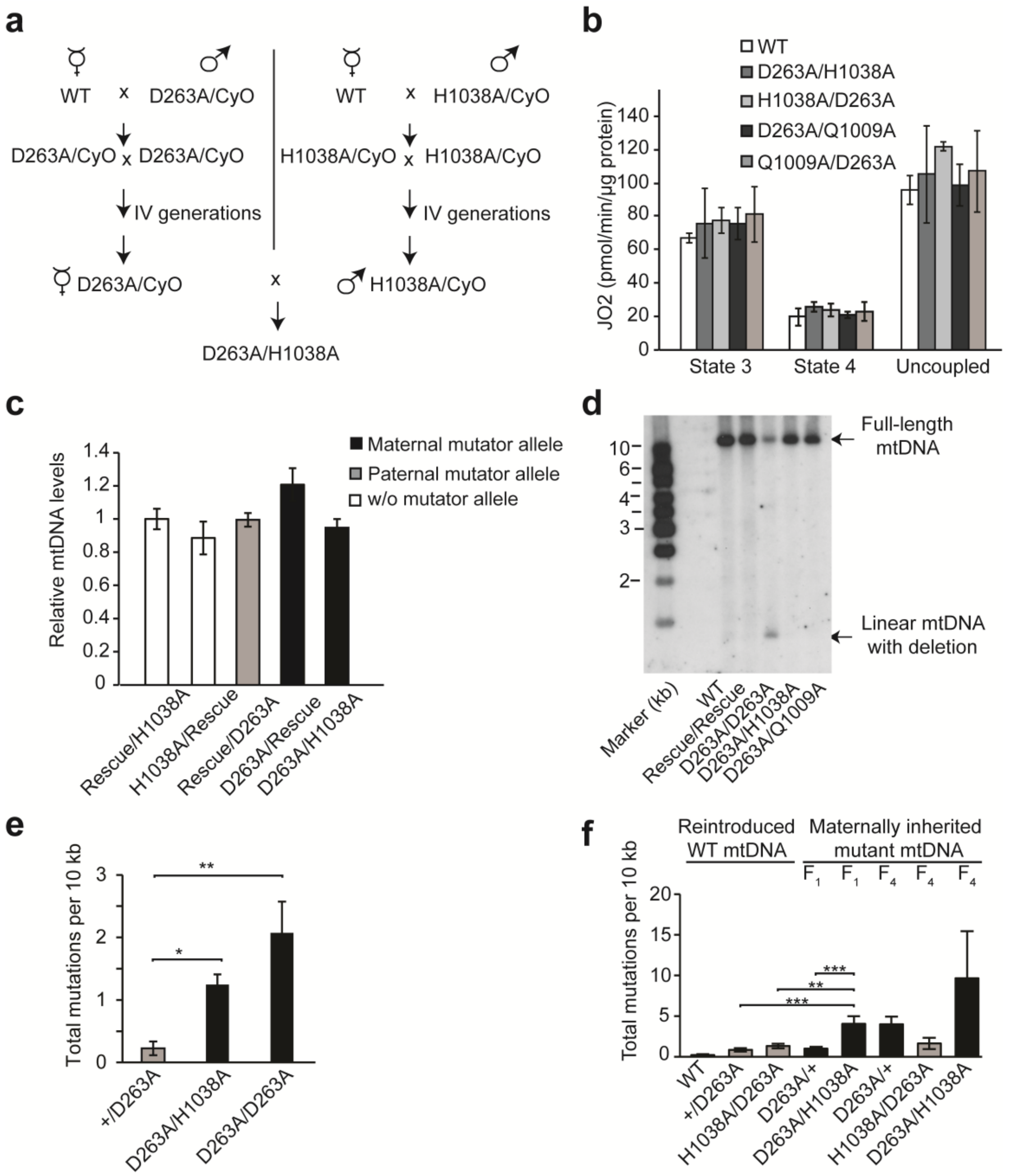

Supplementary Figure 7. Phenotypical analysis of *DmPOLγA* compound heterozygous flies.

(**a**) Crossing scheme to produce *DmPOLγA* compound heterozygous flies with high levels of maternally inherited mutations. Heterozygous genomically engineered *DmPOLγA* flies were intercrossed for 4 generations before the compound heterozygous flies were generated.

(**b**) Respiratory chain function was not affected in *DmPOLγA* compound heterozygotes. Oxygen consumption rates of 5-day-old larvae were measured under phosphorylating (state 3), non-phosphorylating (state 4) and uncoupled conditions and normalized to total protein content. Mann-Whitney test, two-tailed. Error bars represent S.D. ***p<0.001, ** p<0.01, *p<0.05. n=4-6.

(**c**) Steady-state levels of mtDNA in the *DmPOLγA* compound heterozygous flies were determined by qPCR. Total DNA was isolated from adult flies without mtDNA mutations (white bar), with a paternally transmitted D263A allele (grey bar) and with maternally inherited mtDNA mutations (black bar). Data represent two independent experiments. Kruskal-Wallis test with Dunnett's post hoc test. ***p<0.001, ** p<0.01, *p<0.05. Error bars represent S.D. n=5.

(**d**) The linear deleted mtDNA molecules were not present in the *DmPOLγA* compound heterozygous flies. MtDNA was digested with the EcoRV restriction endonuclease and ND2 oligonucleotide was used as a probe. Total DNA was extracted from 5-day-old larvae.

(**e**) Quantification of total mtDNA mutations in compound heterozygous larvae. The homozygous exo- larvae (D263A/D263A) showed tendency to have more total mtDNA mutations in comparison with compound heterozygous larvae with maternally transmitted exo- allele (D263A/H1038A). Tukey's Multiple Comparison test. ***p<0.001, ** p<0.01, *p<0.05. Error bars represent S.D. n=3.

(**f**) Trans complementation between *DmPOLγA* allelic variants caused profound clonal expansion of mtDNA mutations in flies. Compound heterozygous flies, with maternally transmitted D263A allele showed higher

mtDNA mutation loads (black bar) as compared to WT and compound heterozygous flies with a paternally transmitted D263A allele (grey bar). One-way ANOVA with Dunnett's post hoc test. ***$p<0.001$, ** $p<0.01$, *$p<0.05$. Error bars represent S.D. n=3-6.

SUPPLEMENTARY TABLES

Supplementary Table 1. Genetic complementation assay between *DmPOLγA* knockout founder line and deficiency lines.

	Df (2L)Exel7059	Df (2L)BSC252	Df (2L)BSC694
**WT**	50%* (213/425)	49%* (196/404)	52%* (215/413)
**KO**	0%# (0/219)	0%# (0/211)	39%# (73/185)

**Df (2L)BSC252 x Df (2L)Exel7059**	0%# (0/387)
**Df (2L)Exel7059 x Df (2L)BSC694**	0%# (0/68)
**Df (2L)BSC252 x Df (2L)BSC694**	0%# (0/277)

Genetic complementation tests were performed between *DmPOLγA* (*tamas)* knockout line (KO) and deficiency lines that cover (Exel7059, BSC252) or are adjacent (BSC694) to the *tamas* locus. Neither of the deficiencies covering the *tamas* locus (Exel7059, BSC252) was able to complement *tamas* KO, whereas a deficiency adjacent to the *tamas* locus (BSC694) resulted in full complementation (upper table). The deficiencies could not complement each other (lower table). Deficiency lines and *tamas* KO line were kept over a CyO balancer chromosome. (eclosed flies of indicated genotype/total number of flies eclosed) *expected eclosion rate 50%, #expected eclosion rate 25%.

Supplementary Table 2. Viability of allelic complementation groups at the *tamas* locus.

**PolγA alleles**	**Viable as adult fly**
Rescue/Rescue	Yes
D263A/D263A	No
Q1009A/Q1009A	No
H1038A/H1038A	No
Rescue/D263A	Yes
Rescue/Q1009A	Yes
Rescue/H1038A	Yes
D263A/Q1009A	Yes
D263A/H1038A	Yes
Q1009A/H1038A	No

Supplementary Table 3. Genetic complementation assay between *DmPOLγA* mutant alleles and the *DmPOLγA* KO allele.

	Df (2L)Exel7059	Df (2L)BSC252	Df (2L)BSC694
**Rescue**	52%* (368/707)	49%* (232/474)	51%* (299/522)
**D263A**	0%# (0/139)	0%# (0/110)	34%# (156/466)
**Q1009A**	0%# (0/485)	0%# (0/125)	36%# (204/568)
**H1038A**	0%# (0/513)	0%# (0/145)	36%# (113/318)

	TAM3	TAM4
**Rescue**	62% (47/76)	56% (27/48)
**D263A**	0% (0/140)	0% (0/93)
**Q1009A**	0% (0/91)	0% (0/43)
**H1038A**	0% (0/110)	0% (0/59)
**Df (2L)Exel7059**	0% (0/100)	0% (0/64)
**Df (2L)BSC252**	0% (0/95)	N/A
**Df (2L)BSC694**	34% (51/148)	21% (12/56)
**TAM4**	0% (0/71)	

Genetic complementation tests were performed between *DmPOLγA* mutants and deficiency lines that cover (Exel7059, BSC252) or are adjacent (BSC694) to the *tamas* locus (upper table). In addition the hypomorphic *tamas* alleles (tam3 and tam4) were used for complementation assays (lower table). Only the *DmPOLγA* rescue flies were able to complement and they comeplemented all deficiencies as well as both hypomorphic *tamas* alleles. All of the *DmPOLγA* mutants failed to complement deficiencies covering the *tamas* locus or hypomorphic *tamas* alleles. Deficiency lines, TAM3 and TAM4 lines, and *DmPOLγA* mutant lines were kept over a CyO balancer chromosome. (eclosed flies of indicated genotype/total number of flies eclosed) *expected eclosion rate 50%, #expected eclosion rate 25%.

Supplementary Table 4. Developmental analysis of DmPOLγA mutant flies.

Genotype	3rd instar	pupae	adults
wt	91±5	95±1%	95±5%
+/KO	85±6%	78±6%	76±3%
KO/+	90±11%	69±9%	64±11%
KO/KO	13±5%	0	0
D263A/D263A	79±16%	49%	0
Q1009A/Q1009A	43±15%	2±1%	0
H1038A/H1038A	44±17%	0	0
KO/D263A	92±23%	77±24%	19±3%
KO/Q1009A	51±19%	35±18%	0
KO/H1038A	91±12%	38±14%	0
D263A/KO	70±9%	80±14%	24%
Q1009A/KO	58±18%	11±5%	0
H1038A/KO	62±25%	0,7±1%	0

Table shows egg to 3rd instar larval development (3rd instar), 3rd instar larvae to pupae (pupae) and 3rd instar larvae to adult (adult) development. All homozygous DmPOLγA mutant flies developed further than homozygous DmPOLγA knockout flies (KO). A higher percentage of exo- larvae reached the pupal stage compared to the pol- mutants ($p<0.05$). The hemizygous DmPOLγA mutant flies carry one *DmPOLγA* mutant and one *DmPOLγA* knockout allele. Hemizygous DmPOLγA mutants showed improved survival if the pol- allele was transmitted paternally instead of maternally. One-way ANOVA with Dunnett's post hoc test. Genotypes are presented as following: maternally /paternally inherited allele.

Supplementary Table 5. TFAM knockdown flies die during morphogenesis.

%	Eggs	Early Pupae	Late Pupae	Eclosed
**daGAL4/ +**	100	78,4	78,4	77,8
**TFAM RNAi #1/ +**	100	80,8	80,8	80,4
**TFAM RNAi #2/ +**	100	94	94	94
**TFAM RNAi #1/ daGAL4**	100	87,8	80,2	11,2
**TFAM RNAi #2/ daGAL4**	100	79,6	37,6	0

Flies with 65% decrease in mtDNA copy number died mostly in the pharate stage with the presence of some escaper flies (Fig. 5e, TFAM RNAi #1/daGAL4) whereas flies with 85% decrease in mtDNA copy number die mostly after pupariation. Data represent two independent experiments.

Supplementary Table 6. List of primers used in the study.

Primers used to clone POLγA donor constructs for ends out homologous recombination	
**5' homologous arm**	
Pr 1:	atctgcaaacggataggatggttgggttaggaaacacgttatcacgggccgcatgccacaacatacgagccggaagcata
Pr 2:	gtatcggcaacaggatgctttaaatgcaaggttatttaaaaacatagtgaccgcggatgtgcgcggaacccctatttg
**3' homologous arm**	
Pr 1:	tttatagcaaactgaataaaatgttttttattcgtaaaatcaaatgttaaggcgcgcccacaacatacgagccggaagcata
Pr 2:	cagggattgcgagtcccggccatgatcacagccatccagaagcagagctaaggcctatgtgcgcggaacccctatttg
**Primers used for genotyping of genomically engineered DmPOLγA flies**	
**PCR1**	
Pr 1:	tcatttggaatgtggagcag
Pr 2:	aaggagggcatgatcaagaa
**PCR2**	
Pr 1:	cacccgaaattagagctgga
Pr 2:	gaacgcagtggtccagctat
**PCR3**	
Pr 1:	acctgcggtaagtggtcatc
Pr 2:	cactacgcccccaactgagagaac
**PCR4**	
Pr 1:	agaagtgaccgtggagcaac
Pr 2:	ctcgacaccggtataacttcgtataatg
**PCR5**	
Pr 1:	tccaatcccactgactgaca
Pr 2:	aaggagggcatgatcaagaa
**PCR6**	
Pr 1:	tcatttggaatgtggagcag
Pr 2:	gggaataagggcgacacgga
**PCR7**	
Pr 1:	tcatttggaatgtggagcag
Pr 2:	gtggaactgcatcctcgttt
**PCR8**	
Pr 1:	tttctcgagttaagtttgcaaacccttaac
Pr 2:	tttggcgcgccgtgtttgtttttaataattaatcg

Primers for site-specific mutagenesis	
**D263A**	
Pr 1:	cacaatgtctcctacgccagggcgcgactgaag
Pr 2:	cttcagtcgcgccctggcgtaggagacattgtg
**Q1009A**	
Pr 1:	caattgggtggtagcgagcggtgcagtg
Pr 2:	cactgcaccgctcgctaccacccaattg
**H1038A**	
Pr 1:	ctgcttgagcttcgctgatgaattgcgc
Pr 2:	gcgcaattcatcagcgaagctcaagcag

**Primers used to generate the probes for Southern blot analyses**	
**ND2**	
Pr 1:	cttggttaggagcttgaataggt
Pr 2:	aatggaggtaatcctcctaatga
**12S**	
Pr 1:	tcattctaga tacactttccagtacatc
Pr 2:	actaaattggtgccagcagtcgcggt
**COXI**	
Pr 1:	aatggagctggaacaggatg
Pr 2:	tcgaggtattccagccaatc
**Primers used for qPCR analyses**	
**cytB**	
Pr 1:	ttaatcatatttgtcgagacg
Pr 2:	aatgatgcaccgttagcat
**Rpl32**	
Pr 1:	gacgcttaagggacagtatctg
Pr 2:	aaacgcggttctgcatgag
**Primers used for mtDNA mutation load analysis**	
Pr 1:	ttgattttttggtcaccctgaagt
Pr 2:	aacttcaatatcattgatggccg
